# Global Fluoroquinolone Resistance Epidemiology and Implictions for Clinical Use

**DOI:** 10.1155/2012/976273

**Published:** 2012-10-14

**Authors:** Axel Dalhoff

**Affiliations:** Institute for Infection-Medicine, Christian-Albrechts Univerity of Kiel and University Medical Center Schleswig-Holstein, Brunswiker Straße 4, 24105 Kiel, Germany

## Abstract

This paper on the fluoroquinolone resistance epidemiology stratifies the data according to the different prescription patterns by either primary or tertiary caregivers and by indication. Global surveillance studies demonstrate that fluoroquinolone resistance rates increased in the past years in almost all bacterial species except *S. pneumoniae* and *H. influenzae*, causing community-acquired respiratory tract infections. However, 10 to 30% of these isolates harbored first-step mutations conferring low level fluoroquinolone resistance. Fluoroquinolone resistance increased in Enterobacteriaceae causing community acquired or healthcare associated urinary tract infections and intraabdominal infections, exceeding 50% in some parts of the world, particularly in Asia. One to two-thirds of Enterobacteriaceae producing extended spectrum *β*-lactamases were fluoroquinolone resistant too. Furthermore, fluoroquinolones select for methicillin resistance in *Staphylococci*. *Neisseria gonorrhoeae* acquired fluoroquinolone resistance rapidly; actual resistance rates are highly variable and can be as high as almost 100%, particularly in Asia, whereas resistance rates in Europe and North America range from <10% in rural areas to >30% in established sexual networks. In general, the continued increase in fluoroquinolone resistance affects patient management and necessitates changes in some guidelines, for example, treatment of urinary tract, intra-abdominal, skin and skin structure infections, and traveller's diarrhea, or even precludes the use in indications like sexually transmitted diseases and enteric fever.

## 1. Introduction

Nalidixic acid—a byproduct of chloroquine synthesis—was marketed during the 1960s for oral treatment of urinary tract infections and is still available by prescription. Several quinolones were invented since then, including flumequine bearing a fluorine atom at position C-6, which was active against nalidixic acid resistant *Enterobacteriaceae*. However, development of newer fluoroquinolones did not progress significantly till it was demonstrated that substitutions at the C-6 and C-7 positions improved antibacterial activity and pharmacological properties [1]. Since then, fluoroquinolones have become established for treatment of urinary, respiratory, gastrointestinal, urogenital, intra-abdominal, and skin/skin structure infections in outpatients and hospitalised patients. Despite millions of prescriptions in the first two decades of their use, the emergence of quinolone resistance during treatment was uncommon except in *Staphylococcus aureus* particularly in methicillin-resistant *S. aureus* and *P. aeruginosa*. Resistance to fluoroquinolones emerged rapidly in these two species, predominantly due to clonal spread among nursing home residents and immunocompromised patients [2]. However, since the mid 1990s quinolone resistance started to increase in almost all Gram-positive and Gram-negative species and minimal concentrations (MICs) inhibiting 90% of the strains studied varied species specifically over a broad range from ≤0.015 up to ≥128 mg/L [3–5] thus indicating that resistant subpopulations were frequent already two decades ago but passed almost unnoticed. Recent surveillance studies demonstrate that resistance rates continue to increase thus affecting patient management and necessitating a change in some current treatment guidelines [6, 7], or even precluding the use of fluoroquinolones in certain indications as will be discussed below [8, 9].

This paper summarizes data from local, national, international, and global surveillance studies of antimicrobial resistance combining the complementary approaches of routine surveillance (the active investigation of results generated in the course of routine clinical care) and targeted surveys (one-time or periodic study protocols to address specific scientific or public policy needs not adequately addressed by routine diagnostic test results). Data generated in the course of global, longitudinal surveillance studies are complemented with national and regional data. Only those studies using standardized test methods and defined susceptibility-/resistance-criteria according to national—or preferably CLSI—(formerly NCCLS) breakpoint definitions were selected. Many articles quoted in this paper originate from the author's files; others were chosen from searches on Pubmed. Articles summarized in recent reviews were excluded from this synopsis. 

Large global surveillance studies comprising centers in Asia, Asia/Pacific region, Japan, North, Central, and South America, and the EU have the strength that large numbers of pathogens are sampled and that standardized methods of data collection, susceptibility testing and data interpretation are used. Therefore, surveillance programmes like *SENTRY* (a global, longitudinal study on the susceptibility of pathogens causing blood-stream infections, community-and hospital acquired RTIs, skin and soft tissues, and UTIs, sponsored by Bristol Meyers Squibb, recently switched to a study on the susceptibility of Gram-positive pathogens to daptomycin and comparators), *MYSTIC* (meropenem yearly susceptibility test information collection, a global, longitudinal surveillance study designed to evaluate the prevalence and in-vitro antimicrobial susceptibility of isolates from intensive care units, neutropenia units, cystic fibrosis units, or non-specialist centres where meropenem is used, sponsored by Astra Zeneca), *SMART* (study monitoring antimicrobial resistance trends, a study on the susceptibility of intra-abdominal aerobic and anaerobic clinical isolates, sponsored by Merck), *PROTEKT* (prospective resistant organism tracking and epidemiology for the ketolide telithromycin, sponsored by Aventis Pharmaceuticals), *GLOBAL* (global landscape on the bacterial activity of levofloxacin) and the “*Alexander Project*” (an international study that began in 1992 and involved initially 6, later 27 countries, sponsored by GlaxoSmith Kline), data from major European programmes (e.g., European Antimicrobial Resistance Surveillance System (*EARSS*); *ECO. SENS* (*E. coli* sensitivity)) and national programmes (e.g., *NAUTICA* (North American Urinary Tract Infection Collaborative Alliance; the National Nosocomial Infections Surveillance System (*NNIS*)/National Healthcare Safety Network (*NHSN*) established by the Centers for Disease Control and Prevention in the US) are used as one major source of information. The second source of information constitute national or regional studies meeting the above mentioned criteria. The scope and design as well as the strengths and weaknesses of surveillance studies have been critically reviewed previously [10–12]. 

## 2. Mode of Action and Mechanisms of ****Resistance

### 2.1. Interaction with Bacterial Type II Topoisomerases

Fluoroquinolones are the only class of antimicrobial agents in clinical use that are direct inhibitors of bacterial DNA synthesis. Fluoroquinolones inhibit two bacterial enzymes, DNA gyrase and topoisomerase IV, which have essential and distinct roles in DNA replication. The quinolones bind to the complex of each of these enzymes with DNA; the resulting topoisomerase-quinolone-DNA ternary complex subsequently leads to the generation of double-stranded breaks in DNA and blocks progress of the DNA replication enzyme complex. Ultimately, this action results in damage to bacterial DNA and bacterial cell death [13–16].

Resistance to quinolones occurs by mutation in chromosomal genes that encode the subunits of DNA-gyrase and topoisomerase IV (altered target mechanism), and that regulate the expression of cytoplasmic membrane efflux pumps or proteins that constitute outer membrane diffusion channels (altered permeation mechanism). Several excellent and comprehensive reviews have been published summarizing the current knowledge about the mode of action and resistance mechanisms of fluoroquinolones; the reader is kindly referred to these publications for further reading (e.g., [16–21]). Furthermore, reduced target expression has been described as another mechanism leading to low level quinolone resistance [22].

### 2.2. SOS Response and Autoinduction of Fluoroquinolone Resistance

Repair mechanisms are activated as a consequence of inhibition of bacterial type II topoisomerases. Any DNA-damage triggers the production of various repair proteins by activating an SOS gene network [23–27]. The SOS system is composed of more than 40 genes and is controlled by regulatory proteins RecA and LexA. RecA provides a signal for induction of SOS response, while LexA functions as a repressor; binding the gene repressor LexA unmasks its autoproteolytic activity, so that the 40 SOS genes are no longer repressed. The LexA binding site is located in the sequence upstream from *qnrB* (but not *qnrA* or *qnrS*), so that *qnrB* is regulated by the SOS-system, too, in response to DNA damage [28]. In addition, it has been shown recently that the SOS response promotes *qnrB* expression [29]. The peptide QnrB protects bacterial DNA-topoisomerases from quinolone inhibition and provides low-level quinolone resistance (see below Section 2.3. “plasmid mediated fluoroquinolone resistance”). The Qnr-determinants facilitate the emergence of high-level resistance. In *E. coli*, this latter effect depends on the increased mutation ability conferred by the nonessential polymerases Pol II, Pol IV, and Pol V on LexA-cleavage-mediated derepression of their respective genes (*polB*, *dinB,* and *umuDC*; 106). Thus, *qnrB*-mediated quinolone resistance and increased mutation ability are two events triggered by the same signal, namely, the SOS response. Quinolone resistance gene *qnrB* is upregulated by ciprofloxacin in a RecA/LexA-dependent manner, so that quinolone resistance development is an integral part of their mode of action in *qnrB* harboring bacteria. Ciprofloxacin resistant mutants could be elicited much more frequently in LexA positive wild-type strains than in LexA mutant strains [30, 31]. Vice versa, preventing LexA cleavage renders bacteria unable to evolve resistance to fluoroquinolones [30, 31]. Furthermore, SOS response induces persistence to fluoroquinolones [32]. These results support the notion that fluoroquinolones are not only mere selectors of resistant variants but that bacteria themselves play an active role in the mutation of their own genomes. Quinolone resistance is not only acquired via target site mutations, but also via the SOS system by derepression of genes whose products increase mutation rates. In general, interference with bacterial stress response may reduce the emergence of resistance [33]. Furthermore, it was shown recently that ciprofloxacin stimulated SOS independent recombination of divergent DNA sequences in *E. coli*. Thus, fluoroquinolones increase genetic variation via a second, SOS independent mechanism [34]. This mechanism, too, may favour acquisition, evolution, and spread of resistance determinants.

Not only DNA damaging agents like quinolones trigger the SOS response. Beta-lactams interfering with penicillin binding protein 3 [35, 36], zidovudine or trimethoprim [37], and rifampin [30] activate the SOS gene network as well. These data demonstrate that induction of SOS response by any of these drug classes facilitates persistence and evolution of resistance in general. Thus, it may be speculated that these agents, too, may affect quinolone activity and/or resistance development via the SOS promoted expression of *qnrB*. Furthermore, the SOS system contributes to the spread of antibiotic resistance by promoting horizontal dissemination of antibiotic-resistance genes [38] or mutations.

### 2.3. Plasmid Mediated Fluoroquinolone Resistance

The genetic information for target site or efflux resistance mechanisms is commonly chromosomally encoded. However, the emergence of plasmid-mediated and thus transferable fluoroquinolone resistance has also been reported; several mechanisms are known: 1. Qnr, 2. Aminoglycoside acetyltransferase AAC(6′)-Ib-cr, 3. OqxAB, QepA [39–44]. 

The emergence of plasmid-mediated quinolone resistance was first found in strains of *Klebsiella pneumoniae* in one region of the United States in 1998 [45] and shown to be due to a member of the pentapeptide repeat (PPR) family of proteins Qnr (later named QnrA). In the following years, several distantly related plasmid mediated Qnr determinants were described in Enterobacteriaceae (QnrB, QnrC, Qnr D, QnrS) [46, 47]. They have been identified worldwide and are almost always associated with the production of expanded spectrum *β*-lactamases [48–50]. Qnr-like peptides (sharing an amino-acid identity with QnrA of 16 to 22%) have been found in the Gram-positive bacteria *Mycobacterium tuberculosis*, *M. smegmatis*, and *M. avium *[51], *E. faecalis* [52], and in *E. faecium*, *Listeria monocytogenes*, *C. perfringens*, *C. difficile* [53]. Recently, a new chromosomally encoded quinolone resistance gene of the PPR family has been identified in *Stenotrophomonas maltophilia* and has thus been named Smqnr [54]; a *smaqnr* gene has been found in *Serratia marcescens* [55].

Qnr interacts with DNA-gyrase and topoisomerase IV to prevent quinolone inhibition [39, 56]. Qnr protein causes nalidixic acid resistance and reduced susceptibility to or low-level fluoroquinolone resistance [56]. *Qnr*-genes have been found in ciprofloxacin-susceptible isolates as well as quinolone resistant isolates, suggesting that their presence promotes higher level resistance due to chromosomal mutation, as has been shown in the laboratory. Therefore, the presence of *qnr* genes in clinically relevant species of both, Gram-positive and Gram-negative bacteria may foster quinolone resistance development. Furthermore, *qnrA* and *qnrB* genes are usually integrated into integrons which harbor other antibiotic resistance genes such as *β*-lactamases or aminoglycoside inactivating enzymes. Although *qnrS*-genes are not harbored by integrons, they are associated with transposons containing TEM-1 type *β*-lactamases [57]. Consequently, the association of genes encoding for quinolone resistance and resistance to other drug classes like *β*-lactams and aminoglycosides favour the selection and dissemination of fluoroquinolone resistant strains by chemically unrelated drug classes, and vice versa, of *β*-lactam or aminoglycoside-resistant strains by fluoroquinolones (the close correlation between extended spectrum *β*-lactamases (ESBL) production and quinolone resistance is discussed in the chapters on fluoroquinolone resistance). 

*Qnr* genes were also found on the chromosome of an environmental water bacterium, *Shewanella algae*. Other *qnr* homologs have been found in the genome sequences of several *Vibrio *spp. and *Photobacterium profundum* suggesting that water-borne *Vibrionaceae* may have been the source of and may constitute a reservoir for the *qnr* genes [58–60]. Recently it was demonstrated in vitro that the plasmid borne *Shewanella algae qnr* gene could be transferred to Enterobacteriaceae [58].

Another plasmid-encoded quinolone resistance determinant was identified, a variant of the *aac(6 *′*)Ib* gene encoding an aminoglycoside acetyltransferase. The bifunctional aminoglycoside and fluoroquinolone active variant AAC(6′)-Ib-cr catalyzes acetylation of both drug classes [61]. The variant enzyme has acquired the ability to acetylate ciprofloxacin and norfloxacin and reduces ciprofloxacin's activity fourfold [62, 63]. Moxifloxacin and levofloxacin are not acetylated due to the absence of a piperazinyl substituent at position C-7. Interestingly, the first ciprofloxacin resistant clinical isolate (*S. marcescens*) was isolated from a patient treated in the pre-quinolone era with a *β*-lactam and an aminoglycoside; the pre- and post therapy MICs of ciprofloxacin were 0.06 and 4 mg/L, respectively. This strain produced an aminoglycoside acetyltransferase and showed changes in the outer-membrane composition [64]. AAC(6′)-Ib-cr may be more widespread than Qnr-determinants. Both, Qnr- and AAC(6′)-Ib-cr-production are associated with the ESBL production, thus, representing a second mechanism of co-selection of drug-resistance due to exposure to chemically unrelated agents. 

Most recently, a third type of plasmid-mediated quinolone resistance has been identified: the quinolone efflux pumps OqxAB and Qep, [42–44, 65, 66]. The OqxAB- and QepA-proteins confer resistance to hydrophilic fluoroquinolones like norfloxacin, ciprofloxacin, and enrofloxacin, causing a 32- to 64-fold increase in MICs [65–68]. QepA extrudes in addition to quinolones a narrow range of agents such as erythromycin, ethidium bromide, and acrifliavine; OqxAB exports a wider range of agents like ethidium bromide, tetracyclines, chloramphenicol, trimethoprim, olaquindox, and the desinfectants like triclosan [57, 68, 69]. The problem is that the *qepA* gene and an aminoglycoside ribosome methyltransferase are part of a transposable element [66], so that there is a potential of selection of QepA determinants by aminoglycosides and vice versa aminoglycoside resistance by quinolones; the same holds true for *aac(6 *′*)Ib* gene mediated resistances. Extrusion of chemically unrelated agents by efflux-pumps represents a third mechanism of cross-resistance. In conclusion, fluoroquinolone resistance can emerge even in the absence of exposure to this drug class as several coselection mechanisms favour the emergence of quinolone resistance. 

Additional, unknown mechanisms of quinolone resistance must exist as known chromosomally-and plasmid-mediated resistance mechanisms plus the presence of the multidrug efflux pump AcrAB were detected in just 50–70% of high-level quinolone resistant *E. coli* clinical isolates with MICs up to 1,500-fold higher than expected [70]. 

### 2.4. Additional Resistance Mechanisms

Any antibacterial agent interacting with an intracellular target must traverse the bacterial cell-wall and cytoplasmic membrane to reach the target. Once taken up, most antibacterials are actively effluxed. Therefore, fluoroquinolones, too, are affected by permeation barriers and efflux pumps, either in association with target modifications or on their own.

As mentioned above, many Gram-positive and Gram-negative fluoroquinolone-resistant mutant strains do not show any mutation in the quinolones resistance determining region (QRDR). For example, 70% of *E. coli* mutants recovered from besifloxacin selection plates were characterized by the absence of classical QRDR mutations [71] and 61% high-level ciprofloxacin-resistant isolates of *E. coli* accumulated lower levels of ciprofloxacin than the wild type, in addition to the *gyrA* mutations found in all of them [72]. Furthermore, chemically unrelated substances like cyclohexane, salicylate, and tetracycline affected fluoroquinolone susceptibilities of *E. coli*, too; 21 of 57 high level fluoroquinolone-resistant clinical isolates of *E.coli* showed tolerance to cyclohexane, suggesting an elevated broad spectrum efflux activity [73]. Multiple antibiotic resistance (*mar*) genes cause an efflux of a variety of chemically unrelated compounds including different drug classes of antibacterials [74] and are affected by a variety of chemically unrelated substances. The *mar* genes regulate accumulation and thus intracellular concentrations of quinolones by altering the expression of porins and efflux pumps [72, 74]. Another efflux pump, AcrAB, extrudes quinolones out of the bacteria. The pump is partly controlled by the *mar* gene and appears to be the major mechanism of resistance for *mar* mutants [75]. Salicylate and tetracycline induce MarA production, a positive regulator of acrAB transcription, so that salicylate stimulates fluoroquinolone resistance selection. Resistance may be seen with mar expression alone or in combination with type II topoisomerase mutations [74]. The combination of AcrAB overexpression with topoisomerase mutations causes high level fluoroquinolone resistance; over 60% of high-level ciprofloxacin-resistant isolates had an increased production of AcrA [76–78].

Additional nontopoisomerase resistance mechanisms that are not under *mar* control can change quinolone resistance patterns. The *nfxB* gene codes for an altered outer cell membrane protein F, thereby decreasing quinolone entry into the cell [79]. In addition, *soxRS* gene products, which are involved in bacterial adaptation to superoxide stress, affect fluoroquinolone activity, too [73]. 

Various combinations of target enzyme alteration, diminished antibiotic accumulation, and efflux are often seen in fluoroquinolone-resistant *E. coli*, other Enterobacteriaceae and nonfermenters [72, 80]. Cross-resistance between fluoroquinolones and antibacterials of chemically unrelated drug classes is associated with the increased expression of efflux pumps because of their limited substrate specificity. For example, MexAB confers resistance to nonfluorinated and fluoroquinolones, tetracycline, and chloramphenicol, Mex CD confers resistance to fluoroquinolones, erythromycin, trimethoprim, and triclosan, Mex EF confers resistance to the latter plus chloramphenicol, imipenem, and triclosan, and Mex XY confers resistance to fluoroquinolones, erythromycin, and aminoglycosides. Several comprehensive reviews have summarized the impact of fluoroquinolone-extrusion and resistance [80–83]. Consequently, a fluoroquinolone resistant or even multidrug-resistant phenotype can easily be selected by an exposure to a broad range of chemically unrelated drug classes, thus, representing the fourth type of cross-resistance.

These examples illustrate the complexity of fluoroquinolone resistance mechanisms, selection by fluoroquinolones and coselection of resistance by chemically unrelated classes of antibacterials and antiseptics. 

## 3. Fluoroquinolone Resistance Epidemiology

### 3.1. Urinary Tract Infections

The first quinolone used clinically, that is, nalidixic acid, was classified as an “urinary antiseptic;” previous nonfluorinated quinolones were almost exclusively used for treatment of lower urinary tract infections (UTIs). The fluorinated quinolones are characterized by more marked antibacterial activity against uropathogens, so that ciprofloxacin resistant *E. coli* strains isolated from female outpatients were almost nonexistent (<1%) till the mid-1990s; resistance to ciprofloxacin increased slowly from 1.2% in 1998 to 2.5% in 2001 [84]. The same holds true for uropathogenic *E. coli* isolated from male and inpatients, respectively, with a trend towards higher resistance rates among elderly patients [85, 86]. However, the NAUTICA (North American Urinary Tract Infection Collaborative Alliance) study revealed that ciprofloxacin resistance increased to 5.5% in 2004 [87]. Likewise, uropathogens studied between the years 1996 and 2009 in the province of British Columbia demonstrated an increase in fluoroquinolone resistance. The resistance rates in *E. coli* and *K. pneumoniae* increased from <2% in 1996 to ≥20% in 2009; the resistance rates of fluoroquinolones for *P. mirabilis* remained almost constant throughout the years at ≤2%. *Enterococci* demonstrated frequently resistance against fluoroquinolones although resistance rates decreased between 2002 and 2009 [88].

#### 3.1.1. Community Acquired Urinary Tract Infections

Data summarized in Table 1 demonstrate that fluoroquinolone resistance ranges from 2.2% to 69% for strains isolated from patients with uncomplicated, community acquired UTI (CAUTI) and even up to 98% for strains from patients with complicated CAUTIs. Likewise, ESBL production ranged from 2.6% to 100%. Both, fluoroquinolone resistance and ESBL production were highest in the Asia-Pacific region and moderate to low in Europe and North America. The clonality of the isolates has rarely been examined, although high numbers of ESBL producers may indicate that a few clones may predominate amongst the isolates studied (see below, Section 3.1.3). Furthermore, data summarized in Table 1 indicate that on the one hand the *relative* numbers of ESBL producers per centre is high whereas on the other hand the *total* numbers of isolates is still quite small. For example, 100% of the ESBL positive strains were fluoroquinolone-resistant; but this corresponds to 11.8% of the total number of isolates studied [89]. The high relative figures of fluoroquinolone resistant ESBL-producers—which are often mentioned in the abstract instead of the total numbers—may mask the prevalence of fluoroquinolone resistance in uropathogens.

The risk for acquisition of CAUTIs caused by ESBL-positive *E. coli* and the distribution of the ESBL enzyme types was determined in a prospective cohort study [90]. A total of 510 patients with CAUTIs caused by Gram-negative bacteria were included in the study. ESBL producers were detected in 6.3% of uropathogenic *E.coli* isolated from uncomplicated UTIs and 17.4% of *E.coli* isolates from complicated UTIs (*P* < 0.001), most of which (90.2%) were found to harbour CTX-M-15. According to multivariate analysis, more than three urinary tract infection episodes in the preceding year (OR 3.8, *P* < 0.001), use of a  *β*-lactam antibiotic in the preceding 3 months (OR 4.6, *P* < 0.001) and prostatic disease (OR 9.6,  *P* < 0.004) were found to be associated with ESBL positivity. The percentages of isolates with simultaneous resistance to trimethoprim-sulphamethoxazole, ciprofloxacin, and gentamicin were found to be 4.6% in the ESBL negative group and 39.2% in the ESBL positive group (*P* < 0.001) [90]. 

Comprehensive reviews of the worldwide emergence of ESBL producing Enterobacteriaceae indicate that 1st their numbers increase continuously, 2nd ESBL production is diverse, scatters geographically, and originates from both, community associated-as well as healthcare associated infections [91–93], 3rd most of the community isolates are multi-resistant, [92, 94], 4th many isolates are often genetically related and clonal spread has been reported frequently [95–101], 5th the pandemic multiresistant, community associated clone ST 131 is highly prevalent and contributes to 30% to 60% to all fluoroquinolone resistant *E*. *coli* [93, 102]. 

Clearly, the continuously increasing prevalence of ESBL-producing Enterobacteriaceae isolated from out-patients is alarming. However, several studies indicate that the pevalence of ESBL-producing, fluoroquinolone-resistant CAUTI-pathogens may be low. The ECO. SENS (*E. coli* sensitivity) project is a Pan-European survey of the antimicrobial susceptibilities of pathogens from uncomplicated UTIs. Data published in 2003 demonstrate that overall ciprofloxacin resistance in the 2,478 *E. coli* strains collected amounted to 2.3%, ranging from 0% in Austria and Sweden to 5.8% and 14.7% in Portugal and Spain, respectively. Ciprofloxacin-resistance rates in *P. mirabilis*, *Klebsiella* spp. and other *Enterobacteriaceae* were 2.1%, 1.0%, and 0.8%, respectively [103]. The ARESC (Antimicrobial Resistance Epidemiological Survey on Cystitis) study revealed that in uropathogens collected in nine European countries and Brazil from 2003 to 2006 ciprofloxacin resistance in *E. coli* was recorded in >10% of all the isolates in Brazil, Spain, Italy, and Russia; in the remaining European countries, ciprofloxacin resistance ranged from 1.4% in France to 6.7% in Poland [104–106]. As national parts of the ARESC study, 335 and 650 uropathogens, respectively, were isolated most recently from German and Spanish patients with uncomplicated cystitis; fluoroquinolone resistance amounted to 7.7% and 11.9%, respectively [107, 108], thus, indicating that fluoroquinolone resistance did not increase as compared to the previous study period. ESBL production was neither specified in the ECO. SENS nor the ARESC study. 

#### 3.1.2. Healthcare Associated Urinary Tract Infections

Fluoroquinolone resistance ranged from 6.3% to 62% in Gram-negative strains and 20% and 100% of the methicillin-susceptible *S. aureus* (MSSA) and methicillin-resistant *S. aureus* (MRSA), respectively, as well as 59% of the *Enterococci* isolated from patients with complicated, healthcare associated UTI (HAUTI) (Table 1). In general, uropathogens from patients admitted to tertiary care hospitals are less fluoroquinolone susceptible than those from out-patients. Clearly, patients admitted to tertiary care hospitals suffer from chronic diseases, urologic surgery, recurrent infectious diseases necessitating antibacterial therapy prior to the actual study, and so forth, so that one or several risk factors favor development of resistance. High rates of fluoroquinolone resistance were found in patients with HAUTIs evaluated in the emergency department [109, 110] and in nursing home residents [111]. Horizontal transmission of one, or few predominating clone(s) in nursing home residents is frequent [2].

#### 3.1.3. Association between Fluoroquinolone Resistance and Production of Extended Spectrum *β*-Lactamases

Although production of extended spectrum *β*-lactamases (ESBLs) was not analysed in these studies, it may well be that the increase in both, fluoroquinolone resistance and ESBL-production, are closely associated [112]. ESBLs gained prominence and started to spread among uropathogens in North America at the time when these surveillance studies have been performed. 

Since the early 1990s, *E. coli* isolates that produce CTX-M type ESBLs have emerged as a serious cause of UTIs in the community [113–116]. *E. coli* strains that produce CTX-M ESBLs, primarily found in community sources, are becoming widely prevalent worldwide [95–97, 113]. For example, in Spain a threefold rise in community-onset UTIs caused by ESBL-producing *E. coli* over a 3-year period from 0.47% (17 of 3,617 isolates) in 2000 to 1.7% (44 of 2,600 isolates) in 2003 was reported, 31% of which (or 0.54% of the total isolates) were resistant to ciprofloxacin [117]. A nationwide study performed in Spain in 2000 revealed that 93% of the ESBL-producing *K. pneumoniae* strains were isolated from inpatients, whereas 51% of ESBL-producing *E. coli* strains were isolated from outpatients [118]. Risk factors for the acquisition of ESBL-producing *E. coli* in non hospitalised patients with uncomplicated urinary tract infections (uUTIs) were diabetes mellitus (odds ratio (OR) = 5.5), previous fluoroquinolone use (OR = 7.6), previous hospital admission (OR = 18.2), and older age in male patients (OR = 1.03) [119]. A prospective cohort study in 510 patients with CAUTIs caused by Gram-negative bacteria revealed that ESBL producers were detected in 6.3% of uropathogenic *E.coli* isolated from uncomplicated UTIs and 17.4% of *E.coli* isolates from complicated UTIs (*P* < 0.001), most of which (90.2%) were found to harbour CTX-M-15 [19]. According to multivariate analysis, more than three urinary tract infection episodes in the preceding year (OR 3.8, *P* < 0.001), use of a  *β*-lactam antibiotic in the preceding 3 months (OR 4.6, *P* < 0.001) and prostatic disease (OR 9.6, *P* < 0.004) were found to be associated with ESBL positivity. The percentages of isolates with simultaneous resistance to trimethoprim-sulphamethoxazole, ciprofloxacin, and gentamicin were found to be 4.6% in the ESBL-negative group and 39.2% in the ESBL-positive group (*P* < 0.001) [90]. As the CTX-M type is most common among the CAUTI pathogens it is conceivable that many of these isolates may be genetically related. More than two thirds of unduplicated *E. coli* strains isolated from patients admitted to nine different Portuguese hospitals in three different regions were ESBL producers; all of the CAUTI pathogens produced the CTX-M-15 type *β*-lactamase. Three quarters of the ESBL producers belonged to one genetic cluster, indicating countrywide dissemination of one single clone [120]. An analysis of selected *E. coli* strains isolated in eight European countries during 2003 to 2006 from patients with uncomplicated cystitis displaying reduced ciprofloxacin susceptibility revealed that 55 different biochemical profiles could be distinguished; although this finding indicates a substantial heterogeneity, about one third of all isolates belonged to two clonal groups O25:H4-ST 131 and O15:K52:H1. ESBL production was detected in 8.1% of all isolates, CTX-M-15 being the most common; strains belonging to the two predominant clonal groups had ciprofloxacin MICs of 16 and ≥32 mg/L, respectively [91, 102, 121]. Point source dissemination of ESBL-producers is frequent in patients with uUTIs. *E. coli* ST 131 was the most predominant group and accounted for 23.1% and 46%, respectively, of ESBL-positive isolates overall [91, 102]. Nearly all ST 131 isolates were ciprofloxacin resistant. The intercontinental pandemic spread of the ciprofloxacin-resistant *E. coli* O25:H4:ST 131 clonal group producing CTX-M-15 has been described worldwide in hospital and community settings [122, 123]. The sudden worldwide increase of ESBL-producing *E. coli* is mostly due to the single CTX-M-15 positive clone ST131; foreign travel to high-risk areas, such as the Indian subcontinent, play in part a role in the spread of this clone across different continents [124]. The isolation of a multidrug-resistant *E. coli* strain of sequence type ST 131 from an 8-month old girl with severe septic arthritis and contagious osteomyelitis and her healthy mother demonstrates that within household transmission contributes to the dissemination of the ST 131 clonal group, too [125]. Furthermore, plasmid-mediated fluoroquinolone resistance determinants including CTX-M-15 were common in areas of high fluoroquinolone consumption [126] and in nursing home residents in whom a single multiresistant clone spread [127].

#### 3.1.4. Risk Factors for and Impact of Prescribing Habits on Emergence of Fluoroquinolone Resistance

The impact of prescribing of ciprofloxacin on the emergence of fluoroquinolones resistance in uropathogenic *E. coli* was analysed in 72 general practices in the west of Ireland. Over a 4.5 year period (from April 2004 to September 2008) susceptibility and prescribing data were collected and analyzed by a multilevel model with ciprofloxacin-resistance as outcome and prescribing as predictor. The analysis revealed that in “mean” practices with one prescription per month ciprofloxacin resistance was low (3%) whereas in practices with 10 prescriptions per month ciprofloxacin resistance amounted to 5.5% [128]. Analogous effects were noted in patients with CAUTI monitored over a 6-year period in Denver, Colo, USA [129]. In 1999, the initial therapy of uUTI was switched to levofloxacin. The prescriptions increased from 3.1 to 12.7 per 1,000 visits; in parallel, fluoroquinolone resistance increased from 1% to 9%. Risk factors for the acquisition of fluoroquinolone resistant *E. coli* were hospitalization (or for each week of hospitalization = 2.0), and levofloxacin use winthin the previous year (OR 5.6). Similar risk factors were identified by others, too [130–134]. Additional factors favoring the selection of resistant uropathogens are poor adherence to treatment guidelines [135] and dispensing of antibacterials without prescription [136].

Another aspect is worth mentioning and relevant for prescribing policies, hygiene strategies, and resistance statistics. A study on the evolution of quinolone resistance in Barcelona, Spain from 1992 to 1997 revealed that the prevalence of fluoroquinolone resistance in the feces of healthy people was unexpectedly high, 24% in adults and 16% in children, although not used in the pediatric population [137, 138]. The carriage rate was higher than the fluoroquinolone resistance rates among patients with healthcare and community acquired infections (8.3% and 9% in 1992 versus 18% and 17% in 1996, resp.). Increasing fluoroquinolone resistance rates in commensal *E. coli* in children were found in North as well as South America, Africa, and Asia, too [139–145]. Among pediatric blood-stream isolates there was an association between fluoroquinolone resistance and ESBL production [141]. Similarly, the Chinese isolates from pediatric patients are characterized by a high prevalence of plasmid-mediated quinolones resistance; 4.1% were positive for *qnr* and 8.2% for *aac*(*6 *′)*-Ib-cr* genes known to confer low level fluoroquinolone resistance or to inactivate ciprofloxacin, but not moxifloxacin [145]. Isolates from children had relatively high prevalences of ciprofloxacin resistance in the 1990s already although the use of ciprofloxacin in pediatric populations was approved for treatment of inhalational anthrax (post exposure) in August 2000 and for treatment of cUTI in March 2004. The fluoroquinolone resistance in children could be due to the transmission of resistant isolates between adults and children in families, daycare, or school settings and in previous years to the use of fluoroquinolones in poultry populations. These findings demonstrate that spread of fluoroquinolone resistance due to environmental contamination as well as person to person transmission contributes to an increase in the numbers of resistant isolates independent from selection of resistant strains in diseased patients; this phenomenon may bias resistance statistics. Analogues findings will be reported below for RTI-pathogens. Furthermore, these findings indicate that treatment of fluoroquinolone-naïve patients, that is, those who should not have been treated in previous years because of their age, may nevertheless carry primed bacteria which may develop high-level fluoroquinolone resistance quite rapidly during treatment.

*Conclusion*. These data demonstrate that most of the uropathogens causing uncomplicated UTIs in outpatients are still susceptible to fluoroquinolones, but considerable regional differences in drug susceptibility patterns exist with alarming rates of fluoroquinolone-resistant and/or ESBL-producing uropathogens in the Asia-Pacific region and India. Because of the very close correlation between ESBL-production and fluoroquinolone resistance in uropathogenic Enterobacteriaceae, fluoroquinolone susceptibility is still high in all those geographic regions in which ESBL producing Gram-negative community-acquired uropathogens are infrequent. Pathogens causing HAUTIs or cUTIs in nursing home patients are less susceptible to fluoroquinolones. Because of the considerable variability of susceptibility patterns in different countries, local epidemiological data are critical in the empiric management of UTIs, in particular in patients with risk factors and nursing home residents. Furthermore, fluoroquinolones exert a MRSA selective potential and exhibit negative epidemiological effects resulting in the selection of multiresistant pathogens. Therefore, fluoroquinolones should be used with caution even in patients with CAUTI and in particular in patients with HAUTI [146–148].

### 3.2. Respiratory Tract Infections

#### 3.2.1. Community Acquired Respiratory Tract Infections

Although a number of significant pathogens like *Haemophilus influenzae*, *Moraxella catarrhalis*, *Mycoplasma pneumoniae*, *Chlamydia pneumoniae*, and *Legionella pneumophila* are associated with community acquired respiratory tract infections (CARTIs) in all age groups [149–151], *S. pneumoniae* is the most frequent one. In the past, three major RTI surveillance studies, the Alexander Project [152], the RTI component of SENTRY [153], and PROTEKT (prospective resistant organism tracking and epidemiology for the ketolide telithromycin, sponsored by Aventis Pharmaceuticals) [154] have provided invaluable data on global antimicrobial resistance in CARTI-pathogens. Penicillin resistance rates in pneumococci varied from 71% in South Korea, 57% in Hong Kong, and 40% to 50% in France, Spain, and Japan, whereas no penicillin-resistance was detected in Indonesia or the Netherlands [155–161]. Likewise, macrolide resistance among RTI pathogens varied from 0% to 41% [155, 157]. In Taiwan, penicillin and/or macrolide and/or trimethoprim/sulfamethoxazole-resistance amounts to 72%, 92%, and 76%, respectively [162]. Interestingly, even in these “hot spots” of penicillin- and/or macrolide and/or trimethoprim/sulfamethoxazole resistance like Asia or Spain where fluoroquinolone use is high and low doses are administered frequently, rates of fluoroquinolone resistance remain low.

It is important to note that in the studies quoted below the definitions of ciprofloxacin and levofloxacin resistance are based on two different resistant breakpoints, that is, ≥4 mg/L for ciprofloxacin and ≥8 mg/L for levofloxacin. 

No levofloxacin-resistant pneumococci were detected in eight Asian countries from 2002 to 2004 [163, 164]. In Taiwan, only 0.6% of pneumococcal isolates collected from 2000-01 were resistant to levofloxacin [154]; by 2003, 3% of isolates in Taiwan were resistant to levofloxacin [162]. From 192 pneumococcal isolates collected in China from 2001 to 2002, 6.8% were resistant to levofloxacin; 4.2% were resistant to moxifloxacin [160, 165]. In 2008, 6.5% of *S. pneumoniae* isolated from hospitalized patients in Bangkok, Thailand, were resistant to ofloxacin [166]. A national surveillance study in Japan from 1994 to 2002 revealed that levofloxacin resistance rates were below 2% and were stable throughout the observation period; however, an increase in levofloxacin resistance rates from 0% in 1998 to 9.5% in 2000, and 4.8% in 2002 was found among penicillin-resistant pneumococci [161]. Recently, four highly levofloxacin-resistant pneumococci (MIC > 32 mg/L) were detected in Japan among 345 strains collected in Gifu prefecture from May 2006 to July 2006 [167]. Also in Spain, fluoroquinolone resistance rates remain low, ranging from 0.6 to 7% for ciprofloxacin [168–172]. A recent nationwide susceptibility study collected in 34 laboratories 2,559 *S. pneumoniae* isolates from patients with community acquired pneumonia (CAP); only 2.2% and 0.5% of these isolates were ciprofloxacin and levofloxacin resistant [173]. 

Fluoroquinolone resistance is rare in North America. Surveillance studies in the United States from 1987 to 2009 demonstrated low rates of resistance (0.1 to 1.3%) to levofloxacin [174–195] and to moxifloxacin (0.1%; 216). From 27,828 isolates of *S. pneumoniae* collected in the US during 4 consecutive respiratory seasons from 1998 to 2002, only 1.3% were levofloxacin-resistant [181] although ciprofloxacin has been used in the US since 1987 and has thus exerted a selective pressure on *S. pneumoniae*. Likewise, the prevalence of fluoroquinolone resistance in Canada remained low from 1998 to 2009. Although total per capita outpatient use of fluoroquinolones increased during this 10-year period, levofloxacin and moxifloxacin resistance remained unchanged at <2% in the >26,000 isolates collected [196]. However, a trend for rising levofloxacin resistance from <0.5% to >3% was noted in some regions of North America [85, 179, 180, 190, 191]. The GLOBAL (global landscape on the bacterial activity of levofloxacin) surveillance programme is an intiative intended to detect susceptibility changes in CARTI pathogens in Europe and Asia [196]. Results from the programme revealed that the susceptibility profiles of 2,395 *S*. *pneumoniae* isolated from 1997 when the study was initiated till 2007 remained unchanged, that is, ≥96% in Asia and ≥98.6% in Europe [196]. Analogous data were obtained in the course of the Alexander Project, collecting isolates from Europe, Middle East, Asia, South and North America [157, 197]. Likewise, the MOXIAKTIV study (a german multicenter study with 29 participating laboratories) demonstrated that 99.3% of the pneumococci were moxifloxacin and levofloxacin susceptible and the MICs of moxifloxacin were as low as those of the prelaunch isolates [198]. These in vitro findings are mirrored by the low prevalence of fluoroquinolone-resistant strains isolated from patients with pneumococcal pneumonia. In 1.2% of the isolates a first step mutation was detected and 6.7% exhibited an efflux phenotype, despite high fluoroquinolone usage [199]. 

Increasing fluoroquinolone resistance in pneumococci paralleled increased usage of fluoroquinolones in general or 2nd generation quinolones in particular [178, 199–201]. Occasionally, fluoroquinolone resistance resulted in clinical failures in patients with pneumococcal pneumonia having been previously treated empirically with oral fluoroquinolones [160, 185, 202–204]. In total, there were 20 ciprofloxacin and levofloxacin treatment failures reported till January 2005 and reviewed by Fuller and Low [204]. A pretherapy isolate was available in five cases only with MICs ranging from 1 mg/L to 16 mg/L; MICs for the during-therapy isolates ranged from 4 mg/L to >32 mg/L [204]. Thus, the question cannot be answered if resistance may have developed during therapy resulting in clinical failure. This question was recently addressed by Orr et al. [205] who investigated in a tertiary referral hospital in England in 865 patients the incidence and epidemiology of levofloxacin-resistant pneumococci. In six patients a shift towards reduced levofloxacin-susceptibility or -resistance was recorded. Five patients had acquired a new distinct strain and one patient only harboured the same clone [205]. This study revealed that levofloxacin pneumococcal resistance still is uncommon and that in vivo fluoroquinolone resistance development is very rare. If it does occur, strain replacement accounts for the majority of cases. A limitation of this study is that all isolates of *S. pneumoniae* from any body site were eligible for inclusion in the study, irrespective of whether the patient has been treated with a fluoroquinolone or not. Furthermore, hospital guidelines recommend to treat severe community acquired pneumonia with levofloxacin plus intravenous benzylpenicillin [205]. High-level levofloxacin-resistance (MIC > 8 mg/L) developed under levofloxacin-treatment in eight out of 164 patients with chronic obstructive pulmonary disease whose pretherapy isolates were suceptible [206]. A fatal outcome was described in another patient with chronic obstructive pulmonary disease who was infected with a *S. pneumoniae* strain with a preexisting *parC* mutation; the MIC of levofloxacin for this strain was 1 mg/L, so that the mutation passed unnoticed and the strain was classified as susceptible [207]. A *P. aeruginosa* infection was treated successfully with oral ciprofloxacin in another COPD patient in whom a ciprofloxacin resistant but moxifloxacin-susceptible (MIC 0.125 mg/L) *S. pneumoniae* strain was isolated subsequently; this strain harbored a *parC* mutation [208].

The prevalence of first-step fluoroquinolone-resistant *S. pneumoniae* mutants is increasing [195, 200, 208]. Although the subtle changes in MICs of 3rd generation fluoroquinolones for primed bacteria remained within the susceptible range in most CARTI-isolates, many isolates contained a single *gyrA* or *parC* mutation, which prime the bacteria to acquire additional mutations within the quinolone resistance determining region (QRDR) conferring high-grade fluoroquinolone resistance [209–211]. Three up to 30% of clinical pneumococcal isolates contain mutations in the *gyrA* and/or *parC* loci [179, 209, 212, 213].

These data demonstrate that many pneumococcal isolates with first-step fluoroquinolone resistance may pass unnoticed in routine susceptibility testing because of the high resistance breakpoints. This theory has been proven by two in vitro screening tests [214, 215]. Previously, the resistant breakpoints for ciprofloxacin and levofloxacin were >4 mg/L and >8 mg/L, respectively. Actually, the resistant breakpoints of ciprofloxacin and levofloxacin for *S. pneumoniae* defined by EUCAST are >2 mg/L. The EUCAST provides two comments in this context: 1st, wild type *S. pneumoniae* are not considered susceptible to ciprofloxacin, and 2nd the breakpoints for levofloxacin relate to high dose therapy. However, high levofloxacin doses, that is, 750 mg once or 500 mg twice daily, are rarely administered, so that an extrapolation from the categorization “susceptible” due to in vitro breakpoint based susceptibility testing to an advise on therapy in the patient is limited. Two case reports describing levofloxcin treatment failures confirm the limited predictability of routine in vitro susceptibility testing. First, a 71-year-old male patient was hospitalized due to pneumococcal pneumonia. The pretherapy isolate was levofloxacin susceptible with a MIC of 2 mg/L although it had a point mutation in *gyrA*. The patient was treated with 500 mg iv for 13 days; on day 4 intravenous clarithromycin was added and on day 14 treatment was changed. Initial treatment with levofloxacin failed due to an acquisition of a second mutation in *parC* resulting in a MIC of 16 mg/L [216]. Second, a 79-year-old male patient was hospitalized with bacteremic pneumonia caused by levofloxacin susceptible *S. pneumoniae* with a MIC of 1 mg/L. The patient was treated with 500 mg levofloacin iv. After initial improvement fever reappeared on day 4, so that amoxicillin was added; but the clinical condition failed to improve and the patient died one day later. This pathogen had a preexisting mutation in *parC*; the post-therapy isolate had an additional mutation in *gyrA* [207]. Both patients had apart from the advanced age additional risk factors like COPD and others.

These clinical examples confirm that first step mutants of *S. pneumoniae* are 1st phenotypically considered to be susceptible and 2nd are primed to acquire additional QRDR mutations conferring high-grade fluoroquinolone resistance resulting in clinical failure [217]. As most first step mutants pass routine susceptibility testing unnoticed they are not effectively detected in surveillance studies, so that these may be biased. Consequently, routine susceptibility testing of suspicious cases at least should be modified, for example, by using a second fluoroquinolone like ciprofloxacin as an indicator for the acquisition of a first mutation. Furthermore, it should be considered to use a more potent antipneumococcal fluoroquinolone than levofloxacin, for example, a “respiratory fluoroquinolone” like a C-8-methoxyquinolone.

Recently, fluoroquinolone-resistant streptococci were isolated from children. Ciprofloxacin-resistant *S. pneumoniae* were detected in 28% of 847 children of 6 to 60 months of age living in rural Vietnam, about half of which were treated previously with antibacterial agents except fluoroquinolones. This finding could be due to the transmission of already fluoroquinolone-resistant strains within the household from adults to children [218]. Furthermore, ciprofloxacin resistance rates increased significantly (*P* < 0.01) between 1997 and 2006 from 0% to 4.5% in Canadian children aged 0 to 15 years [189]. Elderly are also prone to acquire resistant pneumococci. High fluoroquinolone resistance rates (>10%) were recorded in adults ≥65 years old and in patients who acquired pneumococcal infections in nursing homes [178, 190, 193, 201]. A random sample of surveillance isolates collected in the USA between 1998 and 2003 revealed that 16.2% of isolates were recovered from nursing home patients and 6.4% from non-nursing home patients [219]. 

The emergence of levofloxacin-resistant *S. pneumoniae* strains was noted in South Africa where fluoroquinolones are used to treat multidrug resistant tuberculosis. A survey of 21,521 invasive pneumococcal isolates identified between 2000 and 2006 in South Africa detected levofloxacin-resistance (MIC ≥ 4 *μ*g/mL) in only 12 cases (<0.1%) [220]. All were HIV-infected children; nine were on therapy for tuberculosis; 10 isolates (83%) were serotype 19F, suggesting clonal spread. Furthermore, levofloxacin-resistant pneumococci were detected in >50% of asymptomatic carriers (irrespective of prior exposure to fluoroquinolones). These data suggest that the use of fluoroquinolones to treat multidrug-resistant tuberculosis is a risk factor for endemic and clonal spread of fluoroquinolone-resistant pneumococci. Furthermore, horizontal gene transfer may have transformed low-level into high-level levofloxcin-resistant strains [221]. 

Multiresistant serotype 8 pneumococci (approx. 62% were coresistant to erythromycin, levofloxacin, and tetracycline) causing invasive disease were significantly more frequent in HIV-infected patients than in non-HIV patients admitted to a tertiary care hospital in Madrid, Spain [222], thus indicating that multiresistant pneumococci are a cause for concern in HIV patients.

Despite the global emergence of first- and second step fluoroquinolone-resistant *S. pneumoniae*, the prevalence of resistance in pneumococci isolated from patients suffering from CARTI remained low. Several factors may have contributed to this phenomenon: 1st, more potent “respiratory fluoroquinolones” like the C-8-methoxyquinolones moxifloxacin and gatifloxacin, or gemifloxacin may have replaced the previous fluoroquinolones in the treatment of CARTIs. 2nd, treatment guidelines may have been adapted recommending the use of a second agent like benzylpenicillin in, for example, elderly or patients with other risk factors. 3rd, information about patient history and previous antibiotic use is crucial for determining appropriate empirical therapy [190, 223]. 4th, acquisition of some *parC* and *gyrA* mutations may impose a fitness cost to the first step fluoroquinolone-resistant strains, although equivocal data have been generated [224–226].

*Haemophilus influenzae* is generally highly susceptible to fluoroquinolones; global surveillance studies demonstrated that susceptibility to fluoroquinolones remained at or near 100% [197, 227–230]. Resistant isolates have been recovered occasionally [230–237]. For example, during the 1997 through 1998 SENTRY-programme four (0.13%) fluoroquinolone-resistant *H. influenzae* strains were identified [238]. The strains were genetically distinct and had different *gyrA* mutations. Furthermore, clonal outbreaks of fluoroquinolone-resistant *H. influenzae* were observed in long-term care facilities [239–241] and in elderly in Japan [242]. 

Because of the occurrence of fluoroquinolone-resistant strains, Hirakata et al. [243] screened a total of 400 *H. influenzae* strains isolated in 138 hospitals throughout Japan. The strains were consistently very susceptible to ciprofloxacin with MICs ranging from ≤0.03 to 0.25 mg/L; the majority of strains was inhibited by ciprofloxacin concentrations ≤0.03 mg/L. Therefore, the authors examined the strains (*n* = 37 out of 400) with MICs 0.06 mg/L and higher for QRDR mutations. From these, one ciprofloxacin-resistant isolate (MIC = 16 mg/L) and 31 ciprofloxacin-susceptible isolates (MICs, 0.06 to 0.5 mg/L) had amino acid changes in their QRDRs. Moreover, 9.8% of the 363 highly ciprofloxacin-susceptible isolates (MICs ≤ 0.03 mg/L) had mutations in their QRDRs, particularly in the case of *β*-lactamase positive amoxicillin-clavulanate resistant isolates [243]. 

These data clearly demonstrate that—in analogy to *S. pneumoniae*—many fluoroquinolone-susceptible *H. influenzae* have acquired QRDR mutations; these strains pass routine susceptibility testing unnoticed, but are primed to mutate further. Routine susceptibility testing of suspicious cases at least should be modified, for example, by using nalidixic acid as an indicator for the acquisition of a first mutation [228, 244]. The presence of *H. influenzae* with reduced levofloxacin-susceptibilities in kindergarten children in Hong Kong is alarming; the MICs of nalidixic acid and levofloxacin were 64–128 mg/L and 0.125 mg/L, respectively [245]. Likewise, the report about a levofloxacin treatment failure in a patient with *H. influenzae* pneumonia is worrying. The 71-year-old patient has been treated with 500 mg levofloxacin once daily; after 7 days the clinical condition had not improved and therapy was changed. Levofloxacin MICs for *H. influenzae* isolated from blood-cultures and bronchial aspirates at day 7 amounted uniformly to 16 mg/L and all the isolates had changes in the QRDR [246].

*M. catarrhalis* remains fluoroquinolone susceptible to almost 100%, although resistant strains have been detected in a very few single cases [197, 228, 229, 231, 247]. Two treatment failures with clonally unrelated resistant strains have been reported in patients at risk [248].

*Conclusion*. The three major pathogens causing CARTI are fluoroquinolone-susceptible to almost 100%. However, first-step mutants have been detected frequently not only in treated patients but also in healthy individuals and even children. Such isolates are primed to mutate to high-level fluoroquinolone resistance during subsequent fluoroquinolone-treatment.

#### 3.2.2. Nosocomial Respiratory Tract Infections

In treatment guidelines and reviews, nosocomial pneumonia is further differentiated into healthcare associated pneumonia (HCAP), hospital acquired pneumonia (HAP), and ventilator associated pneumonia (VAP) [249–254]. Bacterial pathogens most frequently associated with HCAP, HAP, and VAP are methicillin-susceptible and -resistant *S. aureus* (MSSA, MRSA), *Pseudomonas aeruginosa*, *H. influenzae*, *K. pneumoniae*, *E. coli*, and occasionally *S. pneumoniae* and *Acinetobacter *spp. [255]. Resistance surveillance studies differentiating the origin of isolates tested according to pneumonia categories are almost nonexistent; resistance-rates are quoted in very general terms even in some of the guidelines quoted above. Therefore, information compiled below summarises susceptibility data for invasive pneumococci or pathogens isolated from sputa obtained preferably from ICU-patients. *S. pneumoniae* isolated from patients with invasive as well as noninvasive diseases in eight European countries and Latin America were examined in the PneumoWorld Study from 2001 to 2003. Susceptibility testing revealed that fluoroquinolone resistance rates ranged from 0% in Austria, Switzerland, and Belgium to 0.9% in Germany and 1.2 to 1.3% in Italy and Portugal [256]. From the bacteraemic pneumococci isolated from 1999 to 2007 in the UK and Ireland, 14.3% were resistant to ciprofloxacin [257]. Rates of levofloxacin-resistance in invasive *S. pneumoniae* collected by the Centers for Disease Control and Prevention (CDC) Active Bacterial Core Surveillance Program Network (ABCS) remained stable throughout the years at about 0.3% to 0.43% [258, 259]. This finding contradicts reports of seven-valent pneumococcal conjugate vaccine-driven expansion of fluoroquinolone resistant clones [164, 260, 261]; others have hypothesized that a decrease in fluoroquinolone resistance among invasive pneumococci may be due to reduction of absolute numbers of isolates within the vaccine serotypes [262]. Nevertheless, the potential for the clonal expansion and dissemination of fluoroquinolone-resistant strains obtained from the ABCS program has been demonstrated [175]. Clonal spread of levofloxacin resistance in invasive *S. pneumoniae* isolates was identified in Madrid, Spain [176]. Likewise, clonal spread of levofloxacin-resistant pneumococci could be demonstrated in strains from Hong Kong, whereas strains collected in Okinawa, Japan, were not clonally related [177]. 

All *S. pneumoniae* blood-isolates sampled in 2005-2006 and 2008 from Canadian emergency room- and ICU patients were ciprofloxacin susceptible [263, 264]. Ciprofloxacin-resistance among MSSA- and MRSA-blood isolates collected in 2008 amounted to 8% and 81.6%; ciprofloxacin-resistance in respiratory isolates was 11%, and 95.6%, respectively [264]. All *H. influenzae* blood-isolates were ciprofloxacin-susceptible [263]. Ciprofloxacin-resistance rates in *E. coli*, *P. aeruginosa*, and *K. pneumoniae* isolated from blood were 21.6%, 16%, and 4.3%, respectively. Eight percent of these *E. coli* isolates were ESBL producers. Ciprofloxacin resistance in respiratory isolates of *E. coli*, *P. aeruginosa*, and *K. pneumoniae* was 31.7%, 18.4%, and 4.5%, respectively [264]. Pathogens isolated from ICU patients not categorized in patients with/without nosocomial RTIs showed variable fluoroquinolone resistance [265]. Pathogens were collected in the USA (283 sites), Canada (87 sites), France (63 sites), Germany (169 sites), and Italy (48 sites) from January 2000 till December 2002. Pneumococci were highly susceptible in all geographic regions. In MSSA and MRSA, fluoroquinolone resistance varied from 4.8% in Canada to 8% in Germany, and from 90.6% in France to 9.6% in Germany, respectively. In *E. coli*, fluoroquinolone resistance ranged from 6.5% in France to 12.7% in Italy; resistance in *K. pneumoniae* ranged from 7.2% in Canada to 9.9% in Italy; resistance in *P. aeruginosa* ranged from 22.9% in Germany to 76.7% in Italy [265]. In ten Asian countries, ciprofloxacin resistances in *P. aeruginosa*, *E. coli*, and *K. pneumoniae* isolated from HAP- and VAP-patients ranged from 4–44%, 26–80%, and 13–68% [266]. Similar rates were reported for Gram-negative species isolated from Indian VAP-patients [267].

Fluoroquinolones have in the past shown good activity against *A. baumannii* [268]; however, over the past decade there has been a constant rise in fluoroquinolone- and multidrug resistance [269, 270]. Fluoroquinolone resistance in *Acinetobacter* spp. isolated from HAP- and VAP-patients in ten Asian countries varied from 23.2 to 92% [250]. Fluoroquinolone resistance in *Acinetobacter *spp. isolates from North American and European ICU-patients with/without nosocomial RTIs ranged from 25.9% in Canada to 76.7% in Italy [265]. Fluoroquinolone resistance in *A. baumannii* isolates sampled from sputa and tracheal aspirates of ICU patients in a tertiary care hospital in Ankara amounted to 86% [271]. 

*Conclusion*. Pneumococci and haemophilia isolated from HCAP, HAP, and VAP patients are almost all fluoroquinolone-susceptible. MSSA and in particular MRSA are frequently fluoroquinolone-resistant. Enterobacteriaceae and nonfermenters are variably fluoroquinolone-resistant, so that the regional resistance pattern has to be considered prior to the use of a fluoroquinolone in the treatment of nosocomial pneumonias.

#### 3.2.3. Cystic Fibrosis

One of the most striking aspects of natural history of *P. aeruginosa* and its association with cystic fibrosis (CF) is the adaptation and heterogeneity exhibited by the organisms as colonisation of the lung develops to a chronic state. In the early stages of colonisation the *P. aeruginosa* population is usually homogeneous with respect to colonial morphology, antigenicity and drug susceptibility. Later, however, considerable heterogeneity is observed and the *P. aeruginosa* population shows a considerable degree of heterogeneous antimicrobial susceptibility with MICs ranging over a broad range from hyper susceptibility to high-level resistance [272–275]. *P. aeruginosa* being heteroresistant to all relevant antibacterials including ciprofloxacin have been described by these authors. For example, the MIC of ciprofloxacin for one genetically homogeneous isolate as determined by routine methods was 0.5 mg/L prior to ciprofloxacin therapy; however, population analysis revealed that hypersusceptible subpopulations were present at high frequencies and subpopulation with MICs up to 16 times the MIC for the entire population were present at frequencies ranging from 2 × 10^0^ to 5 × 10^−2^. The population analysis of the post-exposure isolate showed that the hypersusceptible subpopulations have been eradicated; the subpopulations with 2 to 8 times the pre terapy MIC occurred at frequencies of approx 1 × 10^−2^ and the subpopulations with 32 and 64 times the pre therapy MIC were present with frequencies of 4- and 2 × 10^−4^ [275]. Consequently, there is a high probability in CF patients that multiple subpopulations of *P. aeruginosa* with a broad range of MICs will exist, so that in principle a single MIC value for the entire population does not exist. Therefore, selection of colonies for susceptibility testing [276] as well as routine susceptibility testing of mixed morphotypes of *P. aeruginosa* yields inaccurate results; for example, predictability of ciprofloxacin susceptibility and resistance of a single isolate from a CF patient was 87.0% and 41.7%, respectively [277]. Thus, the value of conventional susceptibility-testing of bacteria isolated from CF patients is questionable [278]. In addition, fluoroquinolone resistance emerges in the first few days of therapy and viable counts of the pathogen are reduced minimally. Therefore, the fluoroquinolone used to treat CF patients must exert pleiotropic effects on *P. aeruginosa*; ciprofloxacin, for example, inhibits quorum sensing [279] or modulates immune response [280, 281]. However, it was demonstrated recently in vitro and in patients that antivirulence interventions based on quorum-sensing inhibition with a macrolide diminish natural selection towards reduced virulence and therefore may increase the prevalence of more virulent genotypes [282]. Thus, it has to be studied clinically in CF patients if a fluoroquinolone may exert quorum sensing inhibition at all, and if the virulence of the pathogen may be affected or not. 

Furthermore, a common feature of *P. aeruginosa* isolated from CF patients is the very high prevalence of mutator (or hypermutable) strains in contrast to those with an up to 1,000-fold lower spontaneous mutation rate of strains isolated from patients with acute infections [283, 284]. Such hypermutator strains persisted and even amplified (50,000-fold) in contrast to nonhypermutator strains despite adequate, that is, administration of standard doses, exposure to ciprofloxacin [285]. Recent studies have shown that mutators may affect modulation of virulence factors, genetic adaptation to the growth environment in the infected patient, persistence and perhaps also transmissibility [286]. 

Conventional susceptibility testing—thus not considering the heterogeneous susceptibility pattern of the subpopulations—of *P. aeruginosa* isolates from CF patients revealed that ciprofloxacin resistance in Europe ranged from 13.7% in Bulgaria [287] to approximately 30% in the UK, Spain, Germany, and Italy [288–291]; 37.4% of the US isolates were ciprofloxacin-resistant [292] Mucoid strains tended to be less ciprofloxacin susceptible than non mucoid isolates [290]; 27.8% of the non mucoid and 35.3% of the mucoid isolates were susceptible to ciprofloxacin. 

Patients with cystic fibrosis suffer from *S. aureus* infections, too. MRSA carriage and infection are becoming increasingly common among CF patients. It appears that healthcare associated-MRSA predominate, but asymptomatic community associated-MRSA colonisation may be a predictor of disease [293]. The emergence and spread of a specific MRSA isolate in Marseille, France, is worrying. This well-adapted multiresistant isolate is closely related to the vancomycin resistant strain Mu50 and spreads rapidly in CF patients [294]. This strain is also characterized by the presence of an antibiotic inducible (e.g., imipenem, tobramycin, ciprofloxacin) bacteriophage which may result in high frequency transfer and the unintended promotion of spread of virulence and resistance determinants.

The presence of hypermutable *P. aeruginosa* and MRSA in CF patients is a threat to the patient and a challenge for any antibacterial agent.

*Conclusion*. *P. aeruginosa* colonising and infecting CF patients are geno- and phenotypically highly heterogeneous, so that any routine susceptibility testing and resistance surveillance studies are misleading. It is an inevitable consequence of therapy that preexisting resistant subpopulations will be selected, so that resistance will develop rapidly under treatment. 

### 3.3. Skin and Skin Structure Infections

Acute bacterial skin and skin structure infections (ABSSSI) are typically monomicrobial and caused by *S. aureus* and *S. pyogenes* which are also the most common pathogens in complicated bacterial skin and skin structure infections (cBSSSI) which are frequently polymicrobial. However, Gram-negative and anaerobic microbes become more prevalent. The most common Gram-negative organisms in cSSSIs include *P. aeruginosa*, *E. coli*, *K. pneumoniae*, and *E. cloacae*. The most common anaerobes isolated are typically *Prevotella*, *Bacteroides*, and *Peptostreptococcus* species [295, 296].

Although *S. pyogenes* were and are still highly susceptible to fluoroquinolones, low incidences (≤8%) of ciprofloxacin resistance have been found globally; fluoroquinolone resistance in Japan is almost nonexistent [297–315]. In Belgium, fluoroquinolone resistance increased from 2.8% to 13.1% from 2003 to 2005 and decreased thereafter to 8.9% in 2006 [307]. It is important to note, that in Belgium approx. 55% of the fluoroquinolone-resistant isolates were recovered from children aged less than 16 years [307]. Although fluoroquinolones are contraindicated in children, ciprofloxacin is often used off-label for select life-threatening conditions. Furthermore, older and thus cheap fluoroquinolones are used topically for treatment of otitis media with otorrhoea through tympanostomy tubes in paediatric patients. 

In the early days of fluoroquinolone development and clinical use the fluoroquinolones were regarded as potential alternatives to MRSA therapy with a *β*-lactam or vancomycin. This was due to the fact that resistance to fluoroquinolones has rarely emerged in the various staphylococcal infection models. Especially in experimental endocarditis caused either by MSSA or MRSA fluoroquinolones proved effective and were not associated with the development of fluoroquinolone resistance in most of the models. In addition, their in vivo activity was equivalent or even superior to that of vancomycin or imipenem [2, 316, 317].

Unfortunately, staphylococci acquire resistance to antibacterials quite rapidly as they are genetically highly variable [318]. The determinant for methicillin resistance is located on the so-called *staphylococcus* cassette chromosome *mec* (*SCCmec*). Some of the *SCCmec* elements contain additional genes for antibiotic resistance encoding for aminglycoside-, tetracycline-, and macrolide-lincosamide-streptogramin-resistance [319, 320]. Furthermore, HA-MRSA tended to develop fluoroquinolone resistance more frequently than MSSA [321, 322]. This phenomenon may be due to the fact that on the chromosomal map of the *S. aureus* genome the *mecA* gene is located between protein A and DNA gyrase genes. Therefore, mutations in the gyrase may have an effect on the expression of mecA in HA-MRSA strains [323] and some cell wall associated proteins such as protein A and fibronectin binding proteins [324, 325]. Thus, almost any antibacterial drug class has a methicillin-resistance selective potential [326–328], so that strains of HA-MRSA are almost always multidrug-resistant. 

Therefore, fluoroquinolone resistance developed rapidly in the early days of fluoroquinolone therapy in HA-MRSA. Hospital admissions in the US for ABSSSI caused by fluoroquinolone resistant MRSA increased from 29% between 2000 and 2004 [329] to 70.3% in 2008 [330]. In addition, fluoroquinolone-resistant HA-MRSA were spread horizontally as were HA-MRSA as such, so that nowadays neither the 2nd-nor the 3rd-generation fluoroquinolones represent alternatives for treatment of HA-MRSA infections [5, 331–338]. 

In recent years, the emergence of CA-MRSA has complicated the treatment of even ABSSSI [296, 332, 333]. CA-MRSA strains differ in several ways from HA-MRSA strains like composition of the SCC* mec*, the carriage of plasmids encoding resistance to antibacterials of other drug classes and in their associated pathogenicity factors [336]. In contrast to multidrug resistance usually seen in HA-MRSA strains, antibiotic resistance in CA-MRSA is most often limited to macrolides [319, 337–340], so that it has previously been proposed that some 3rd-generation fluoroquinolones could be useful in the treatment of CA-MRSA, since the causative pathogens were usually susceptible to even ciprofloxacin [341–346]. But recently mupirocin, tetracycline, clindamycin, and moxifloxacin (and thus to any commercially available fluoroquinolone) resistance development has been reported [347, 348]. The clone USA 300 became the predominant strain type in the USA and has spread to Europe, South America, and Australia [347, 349, 350]. The lineage USA 100 is frequent, too [351]. Fluoroquinolone resistance in isolates recovered from a phase IV study in patients with cSSSI in the USA and EU from 2004 to 2007 was high; 100% of USA 100-isolates and 42.6% of USA 300 isolates were resistant to gatifloxacin [351]. Community MRSA isolates in general, and the USA 300 clone in particular are increasingly multidrug resistant, with resistance profiles recently broadening to include clindamycin, tetracycline, mupirocin, and fluoroquinolone agents, in addition to the *β*-lactams; occasionally, community isolates also display reduced susceptibility to vancomycin or resistance to gentamicin or trimethoprim-sulfamethoxazole [352].

Pathogens collected from 27 USA and 28 EU medical centers in 2009 causing cBSSSI were variably susceptible to fluoroquinolones: levofloxacin resistance in the USA/EU amounted to 70.3%/84.1% in MRSA, 11.1%/5.4% in MSSA, 54.2%/52.3% in coagulase-negative staphylococci, 0.9%/0.0% in *β*-hemolytic streptococci, 13.6%/1.1% in viridans streptococci, 37%/29.2% in *E. faecalis*, 24.7%/21.8% in *E. coli*, 11%/13.3% in *Klebsiella *spp., and 20.8%/8.0% in *P. mirabilis* [353]. These resistance rates are within the same range as those reported in the late 1990s and 2001–2004 for Gram-negative and Gram-positive aerobic pathogens isolated in North America, Latin America, and Europe from skin and soft tissues [354–356], thus, indicating that resistance rates did not change substantially over time.

Of 175 anaerobic bacteria isolated in the late 1990s from bacterial skin and soft-tissue infections, 27% were levofloxacin-resistant [357]. All *Peptostreptococcus* species isolated from hospitalised patients with diabetic foot wound infection were susceptible to levofloxacin and moxifloxacin; resistance (5–7%) was found in isolates of *B. fragilis*, *Bacteroides ovatus*, and *Prevotella *species collected in 1999 to 2002. [358, 359]. Against *B. fragilis*, moxifloxacin's MIC^90^ was 1.0 *μ*g/mL. Against other *Bacteroides* species, the MIC^90^ was 2–4 *μ*g/mL. Moxifloxacin was least active against *Fusobacterium* species other than *F. nucleatum* (MIC^90^, 8 mg/L). Among anaerobic species isolated from patients with moderate to severe diabetic foot infections from 2001 to 2004 in the USA, 24% were fluoroquinolone resistant [356]. In detail, moxifloxacin resistance rates were: 43% *B. fragilis* group, 10% *Fusobacterium *spp., 2% *Porphyromonas *spp., Gram-positive cocci 18%, and Gram-positive rods 12% [283]. As levofloxacin is less active against anaerobes, resistance rates were correspondingly higher. Of all infection sites, decubitus ulcer isolates had the highest resistance rates [360].

*Conclusion*. In principle, a 3rd generation fluoroquinolone is well suited for treatment of polymicrobial SSSIs because of its broad antibacterial spectrum. Fluoroquinolone resistance rates among pathogens causing skin and soft tissue infections is low in MSSA, and streptococci, moderate in Gram-negative aerobes as well as Gram-positive anaerobes, and high in CA-MRSA, HA-MRSA, and Gram-negative anaerobes. This heterogenous susceptibility pattern may limit the use of fluoroquinolones in the treatment of ABSSSIs and cBSSSIs. 

### 3.4. Intra-Abdominal Infections

The Surgical Infection Society and the Infectious Diseases Society of America (IDSA) have recently published guidelines for the diagnosis and treatment of complicated intra-abdominal infections (IAIs). *E. coli*, *Enterococcus *spp., *Bacteroides fragilis*, and other *Bacteroides* species are the most common pathogens associated with intra-abdominal infections [7, 361]. Intra-abdominal infections are commonly due to mixed aerobic and anaerobic populations, so that a clinically effective regimen has to cover both, the aerobic *Enterobacteriaceae* and Enterococci, as well the anaerobic bacteria.

Several surveillance studies have demonstrated that there is a global trend toward decreasing susceptibilities of anaerobes to antibacterial agents since two decades. Although the rates of resistance show clinically important variations between continents, countries, and counties, almost all drug classes—except metronidazole—like beta-lactams including the carbapenems, clindamycin and quinolones lose activity against anaerobes. A dramatic loss of antianaerobic activity of fluoroquinolones in particular has been noted, exceeding 50% in some parts of the world [362–367].

The continuously increasing quinolone resistance amongst anaerobes is surprising because of a variety of reasons: First, previous fluoroquinolones like norfloxcin, ofloxacin, ciprofloxacin, levofloxacin were not used clinically for treatment of anaerobic infections. Nevertheless, surveillance testing in the US between 1994 and 1996, that is, prior to the launch of the first antianaerobic quinolone trovafloxacin, revealed that quinolone resistance ranged from 3% to 8%. Second, quinolone resistance rates increased in 1997 to 13%, although trovafloxacin was approved in December 1997. Quinolone resistance continued to increase to 15% in 1998. Despite the limited use of trovafloxacin in 1998 and its relegation to a restricted therapeutic category in June 1999, frequencies of quinolone resistance increased further, peaking at 25% in 2001 [360, 368]. Furthermore, it has been speculated that third, older fluoroquinolones like norfloxacin, ciprofloxacin, ofloxacin, and levofloxacin may have fostered quinolone resistance development [360, 368]. However, this hypothesis is not convincing either as the older fluoroquinolones have been heavily used since their launch. Furthermore, the older fluoroquinolones are almost inactive against anaerobes [369, 370]. Although very high total concentrations are achieved in the faeces, free and thus antibacterial active concentrations, are low as quinolones are highly and tightly bound to cell debris, DNA, cellulose, and other fecal matter [371]; therefore, norfloxacin, ciprofloxacin, ofloxacin, and levofloxacin suppress growth of fecal aerobic Gram-negative rods but do not affect significantly the anaerobic flora. Anaerobes with increased MICs or quinolone resistance have rarely been isolated from patients during or shortly after a quinolone treatment [372]. Thus, the driving forces for quinolone resistance development are at present unknown.

Very heterogeneous data on quinolone resistance amongst anaerobes have been reported, ranging for example from 27% to 50% in the US [363, 373], and 44.4% in Canada [374], 15% to 25% in Spain [275, 364, 365, 375], and 0% to 32% in Greece [364, 365, 376]. This heterogeneity of susceptibility data within and between countries—which is typical for aerobic species, too—may reflect marked differences in 1st, the patient populations from whom the isolates were obtained (health-care versus community acquired infections—which, however, is almost always not specified); 2nd, prior antibiotic exposure; 3rd, the patient populations admitted to either tertiary care-or primary care centres; 4th, the sites of isolation; 5th, the limited number of participating centres. Overall, 9% of the European *B. fragilis* group isolates were moxifloxacin-resistant in 2002; a moderate increase to 13.6% was noted in 2009. Geographical differences were detected in 2009, too, with higher resistance rates for moxifloxacin in Scandinavian (21.4%) and Eastern (11.3%) than in Mediterranean countries (5.4%) [365].

A most recent German study with 32 participating centers revealed that moxifloxacin MICs for anaerobes are by one to two titration steps higher than prior to its launch. Resistance rates ranged from 10% to 22% for various anaerobic species except *B. vulgatus*, with 59% of the isolates being resistant. It became evident, too, that resistance rates are higher in isolates obtained from 1st, tertiary care versus primary care centres, 2nd, patients admitted to the ICU versus standard care, and 3rd, health care versus community acquired infections [377]. 

The resistance epidemiology of quinolone resistance among anaerobes has to be complemented with resistance figures in Enterobacteriaceae isolated from patients with intra-abdominal infections in order to cover the entire spectrum of potential pathogens. In general, the situation in Asia is alarming as resistance-rates surpass 60% of the isolates being resistant to ampicillin-sulbactam or a quinolone and producing ESBL [378–384]. ESBL production in *E. coli*, *K. pneumoniae*, or *K. oxytoca* was highly variable in the Asia-Pacific region ranging in total from 4.4% in New Zealand to 77.4% in India. Only 17% and 27% of the ESBL producing *E. coli* and *K. pneumoniae* strains, respectively, were susceptible to ciprofloxacin [379]. In Europe, 11.8% and 17.9% of the *E. coli* and *K. pneumoniae* strains isolated from patients with intra-abdominal infections were ESBL producers [385], ranging from 0% in Lithuania and Switzerland to 30% in Greece. From these, 70% or 78% and 50% or 70% of the community or hospital acquired *E. coli* and *K. pneumoniae* strains were ciprofloxacin resistant. In the US, ESBL production was detected in 4.7% and 17.5% of *E*. *coli* and *K*. *pneumoniae* isolates, respectively. 

From these, 33% and 19% were susceptible to ciprofloxacin [386]. Ciprofloxacin resistance in a worldwide collection of IAI pathogens amounted to 22.8% in *E. coli*, and 15.6% in *K. pneumoniae* [387]. Both, ESBL production and fluoroquinolone resistance remained high or even increased in 2009-2010 in the Asia-Pacific region, Europe, North- and Latin America; ESBL producers were more frequently isolated from elderly [388–393]. These data confirm—in analogy to the UTI-isolates—the very close correlation between ESBL production and fluoroquinolone resistance in *Enterobacteriaceae* causing IAI. Consequently, fluoroquinolone susceptibility is still high in all those geographic regions in which ESBL-producing Gram-negative bacilli are infrequent. Another clinically relevant finding—again in agreement with UTIs—is that fluoroquinolone resistance was much lower in strains isolated from patients with community acquired intra-abdominal infections than in those from hospital-acquired infections.

*Conclusion*. Fluoroquinolone resistance is high amongst aerobic and anaerobic intra-abdominal pathogens. Therefore, the Infectious Diseases Society of America and the Surgical Infection Society published a guideline in late 2009 recommending that antibacterials to be used in the empiric treatment of even community-acquired intra-abdominal infections including mild to moderate infections should be active against both, aerobic and anaerobic pathogens. Consequently, the use of quinolones should be restricted unless resistance rates are lower than 10% [7, 361, 394].

### 3.5. Sexually Transmitted Diseases

Infections caused by *Neisseria gonorrhoeae* and *Chlamydia trachomatis* are the most frequent ones among reportable bacterial sexually transmitted diseases (STD) gonorrhoea, syphilis, and chancroid. Infections due to *Chlamydia *spp. were diagnosed almost 4-times more frequently than infections due to *Neisseria *spp. (409.2 cases versus 110.7 cases in the USA in 2009). *Chlamydia *spp. diagnosis increased by 2.8% in 2009 as compared to 2008, and by nearly 20% since 2006, likely due to expanded screening. Gonorrhea cases declined by 11% overall. Syphilis cases increased, too, while chancroid cases have declined steadily till 2001 and are fluctuating since then. However, *Haemophilus ducreyi*, the causative organisms of chancroid, is difficult to culture, so that this condition may be substantially underdiagnosed. In general, there were large disparities by age, race, and geographical distribution [395–397]. Pelvic inflammatory disease (PID) is a common and serious complication of some sexually transmitted diseases. Two-thirds of cases are considered to be due to sexually-transmitted infections caused by *N. gonorrhoeae* and *C. trachomatis*; one-third (particularly in older women) are commonly polymicrobial. Other pathogens such as *Mycoplasma genitalium* and bacterial vaginosis pathogens (e.g., *Gardnerella vaginalis*, *Mycoplasma hominis*, *Mobiluncus* spp. and other anaerobes) may cause PID, too. Actinomycetes are part of the normal vaginal flora and a rare cause of PID. Therefore, management of PID must take into account in particular the three major pathogens *N. gonorrhoeae*, *C. trachomatis*, and *M. genitalium*.

Coinfections with *C. trachomatis* and *N. gonorrhoeae* are common among young heterosexual patients with gonorrhea. Therefore, all treatments for STD/PID should cover both, *N. gonorrhoeae* and *C. trachomatis* as well as anaerobes [398, 399], and *M. genitalium* has to be considered [397].

#### 3.5.1. *Neisseria gonorrhoeae*

Initially, *Neisseria *spp. was extremely susceptible to fluoroquinolones with ciprofloxacin MICs of  ≤0.008 mg/L. However, low level resistance (0.06–0.5 mg/L) was reported shortly after its launch [400–402], followed soon by high-level resistance (MICs of ciprofloxacin >1.0 mg/L) associated with treatment failures [402–404]. High-level fluoroquinolone resistance is first, more likely to emerge in areas with a high prevalence of low-level resistance; second, it is spread intercontinentally by travellers and an intercity spread and transmission has been reported; third, mono-as well as multi-clonal spread of quinolone-resistant isolates has been reported [405–407]. 

Typically, several different strain types can be identified by using molecular typing methods; for example, 24 different quinolone-resistant strain types were identified among the isolates having caused an outbreak in California, but only four of these were considered outbreak types and comprised 66% of all the isolates [408]. Furthermore, importation (often repeated importation) of one or a few clone(s) and ultimate introduction into established sexual networks have caused the emergence and spread of resistant gonococci rather than de novo emergence as a result of selection by quinolone use or misuse [409]. 

Both, low-level and high-level fluoroquinolone resistance has been reported from all parts of the world (reviewed in [410]). Ciprofloxacin resistance in *N. gonorrhoeae* is highest in Asia; resistance rates in China vary from 40 to 100%, depending on the region studied [410–413]. In Korea, ciprofloxacin resistance increased from 9% in 1992, to 84% in 1999, and to 90.5% in 2004 and 83% in 2006 [414, 415]. In India, ciprofloxacin resistance varied from 80.7% in 2002, 97.2% in 2004 to 88.6% in 2006 [416–418]. In Pakistan, ofloxacin-resistance increased from 0% in 1998 to 92.5% in 2009 [419] and ciprofloxacin resistance in isolates collected from 2007 to 2010 in Iran amounted to 53.2% [420]. In Kenya, ciprofloxacin resistance increased from 9.5% in 2007 to 50% in 2009 [421] and ranged in other African countries from 0% in Malawi or Mozambique to 41.9% in South Africa [422]. Quinolone resistance in the Western Pacific Region ranged in 2009 from ≤1.5% Fiji, Papua New Guinea and New Caledonia via 35% to 42% in New Zealand and Australia up to >95% in Vietnam, Philippines, and Hong Kong, [423]. Gonococcal resistance to ciprofloxacin in the Netherlands, Italy, Greece and in Norway exceed 40% [424–427] which is in the same range as the data previously reported by the “European Surveillance of Sexually Transmitted Infections” (ESSTI) [428] and by the EUROSURVEILLANCE [429]. However, ciprofloxacin-resistance increased to 63% in 17 European countries participating in the European gonococcal antimicrobial surveillance programme, 2009 [430] and was high in the eastern part of the WHO European region, too [431]. Rates of ciprofloxacin resistance amongst the gonococcal isolates rose in Canada from 1.4% in 2001 to 28% in 2006/2007 [432, 433] and the US from <1% in 2001 to 6.7% in the first half of 2006 to 14.8% in 2007, decreasing to 13.5% and 9.6% in 2008 and 2009, respectively, increasing again to 12.5% in 2010 [395, 434, 435]. Consequently, quinolones are not recommended as first-line therapy of *N*. *gonorrhoeae* infections anymore [435–438]. The emergence of multi-drug resistant *N. gonorrhoeae* reduces the treatment-options further [439–444] as such isolates are resistant to quinolones, third generation cephalosporins, and additional agents.

#### 3.5.2. *Chlamydia trachomatis*

Quinolone resistance in *C. pneumoniae* has not been described clinically or even in vitro; however, high-level resistance to ofloxacin, sparfloxacin, and ciprofloxacin occurred in *C. trachomatis* upon serial exposure to subinhibitory quinolone-concentrations [445–449]. However, spontaneous mutation frequencies resulting in moxifloacin resistance were very low or even nonexistent; exposure of *C. trachomatis* serovars L_2_ and D resulted in emergence of quinolone resistance at a frequency of 2.0–2.2 × 10^−8^ in serovar L_2_ only, whereas no resistant clones could be elicited in serovar D [450]. It is important to note that these experiments were performed under routine conditions, that is, a relatively high inoculum (approx. 2.7 × 10^9^ inclusion forming units) was exposed to the drug, whereas the bacterial load at the focus of infection is much lower thus reducing the likelihood of drug-induced resistance selection. Nevertheless, fluoroquinolone-resistant strains of *C. trachomatis* have been isolated occasionally [449, 450]. Fluoroquinolone resistance elicited in vitro in *C. trachomatis* serovar L_2_ was due to a single nucleotide point mutation in *gyrA*, while no mutations were found in *gyrB*, *parC*, or *parE* genes; no QRDR mutations could be detected in the fluoroquinolone-resistant clinical isolates [451].

#### 3.5.3. *Mycoplasma genitalium*

Surveillance studies for antimicrobial-resistance in general and fluoroquinolone resistance in particular are not existent as culturing of this species from clinical specimens is extremely difficult. Acquired resistance to fluoroquinolones has been described in single cases. Analysis of the *gyrA* and *parC* genes of *M. genitalium* isolated from 6 men in whom levofloxacin therapy failed [452] revealed that in one patient a ParC amino acid change could be detected in the pre- as well as post-therapy isolate, whereas in another patient a ParC-mutation was detectable in the post-therapy isolate only. No QRDR mutations could be detected in strains isolated from the remaining four patients [453]. *M. genitalium* clinical isolates from 28 men with nongonococcal urethritis positive for *M. genitalium* were analyzed by PCR. QRDR-mutations were found in five of these 28 isolates; no alterations were detected in the remaining isolates [454]. The two studies quoted above were performed by noncultural methods, so that no MICs could be determined; thus, an association between QRDR mutations and fluoroquinolone resistance and persistence cannot be proven. Furthermore, it should be considered that the patients in whom persisters could be isolated had been treated with low levofloxacin doses (100 mg t.i.d. for 14 days); in addition, levofloxacin is characterized by a moderate activity against *M*. *genitalium* while for example, C8-methoxyquinolones are ten times as active [455, 456].

*Conclusion*. Resistance of *N. gonorrhoeae* to antimicrobials continues to increase worldwide, although considerable geographical variations in resistance exist. Therefore, fluoro-quinolones are not recommended as first-line therapy of *N. gonorrhoeae* infections anymore [435–438]. However, local quinolone-treatment options based on local surveillance data may be reasonable, because of the geographical variations in resistance. All regimens used to treat PID should cover both, *N. gonorrhoeae* and *C. trachomatis*, so that the use of fluoroquinolones in this indication is limited, too [399]. In case parenteral *β*-lactam therapy is not feasible, oral use of fluoroquinolones with or without metronidazole is recommended provided treatment is based on results of antimicrobial susceptibility testing [399].

### 3.6. Traveller's Diarrhea

Enterotoxigenic and enteroaggregative *E. coli* (ETEC and EAEC) are the major causes of bacterial traveler's diarrhea causing up to 80% of acute cases; *Shigella *spp., *Salmonella *spp., and *Campylobacter *spp., as well as viruses and protozoa cause the remainder 20% of cases. Although widely present, the bacterial pathogens show seasonal as well as geographic occurrence patterns [457–460].

In the early days of fluoroquinolone treatment of gastrointestinal infections, ciprofloxacin and other fluoroquinolones were found to be highly active in vitro and clinically effective in the treatment of traveler's diarrhea [461, 462]. However, a study performed during 1997 indicated that the MIC_90_-values of ciprofloxacin and levofloxacin for enteropathogens collected in India, Jamaica, Mexico, and Kenya were as low as 0.125 mg/L and 0.25 mg/L; however, the individual MICs ranged from <0.0156 to 256 mg/L, thus, indicating that fluoroquinolone-resistant strains have emerged already [460]. Another study assessing the evolution of antimicrobial resistance in EAEC and ETEC causing diarrhea in patients who had traveled to different developing countries, comparing two periods of time, 1994–1997 and 2001–2004 revealed that a statistically significant increase in resistance (*P* < 0.01) was observed for nalidixic acid and ciprofloxacin. Mutations in the *gyrA* gene were found in all nalidixic acid-resistant isolates, whereas mutation(s) in both *gyrA* and *parC* genes were found in the ciprofloxacin-resistant isolates. The prevalence of quinolone-resistant EAEC and ETEC was high among the isolates from patients who had travelled to North Africa (50% of EAEC and 43% of EAEC were resistant to quinolones) and among the isolates from patients who had traveled to the Indian subcontinent (66% of EAEC and 28% to 64% of ETEC were resistant to quinolones). In addition, 33% of the ETEC strains from patients traveling to South-east Asia were also quinolone resistant [463–465]. Results for strains isolated from travelers to India [464], Mexico, Guatemala, India [465], and Ghana [466] confirm that fluoroquinolone resistance increased significantly during the past decade. 

Recently, ESBL-producing EAEC were isolated from patients who had traveled to India [467]. Out of 51 EAEC isolates five CTX-M-15 producers were identified which were resistant to fluoroquinolones, too. Three of these five isolates belonged to the same clonal type. ESBL-producing diarrheagenic *E. coli* strains were isolated from children under five years of age in Nicaragua; the ciprofloxacin-MICs ranged up to 8 mg/L [468]. Diarrheagenic *E. coli*, in which, however, ESBL production has not been specified, were isolated from children in Brazil [469] and Vietnam [470]. The isolation of ESBL-producing diarrheagenic pathogens from children suggests that such strains being frequently multidrug-resistant are widespread in the community.

A comparison of the MIC_90_ values of ciprofloxacin for stains isolated in 1997 and 2006–2008 revealed that the susceptibilities of *C. jejuni*, *Salmonella *spp., and *Shigella *spp. remained unchanged, ranging from 0.06 to 0.125 [465]. However, nalidixic acid and ciprofloxacin are frequently used in several parts of the world for empirical treatment of typhoid fever and other enteric infections, so that nalidixic acid-resistance was frequent in the 1990s already; some of the nalidixic acid-resistant strains isolated in India, Jamaica, Mexico, and Kenya were cross-resistant to ciprofloxacin [465]. Resistance to fluoroquinolones increased in enteropathogens other than *E. coli* over the past years causing problems in all regions of the world, including the USA and Europe [471, 472]. However, fluoroquinolone resistance differed by race, ethnicity, age, travel, and species. Only 0.5% of *Shigella *spp. strains isolated in the USA were ciprofloxacin resistant [473]; likewise, none of the *Salmonella *spp. and *Shigella *spp. strains isolated from children under five years with diarrhea in rural Mozambique were resistant to ciprofloxacin [474]. On the other hand, nalidixic acid resistance in *Shigella *spp. and *Salmonella *spp. strains examined in Teheran, Iran, increased from 9.2% in 2001 to 42.3% in 2005 [475], and ofloxacin-resistant *Campylobacter* spp. strains collected over a 11 year period in Pakistan increased from 0% in 1992 to 23% in 2002 [476]. In the UK, an increase of ciprofloxacin-resistant *Campylobacter* spp. from 7% in 1995 to 37.5% in 2008 was reported [477] and 80.5% of the *Campylobacter* spp. strains isolated in five different Portuguese cities over a five year period from 2003 to 2007 were ciprofloxacin-resistant [478]. Plasmid-mediated quinolone resistance is frequent among *Salmonella *spp. and *Shigella *spp. [42–45].

*Conclusion*. The fluoroquinolones have been the most effective antibiotics for the prophylaxis and treatment of bacterial travelers' diarrhea pathogens, but increasing resistance to these agents, mainly among Campylobacter species, may limit their benefit in the future [457, 479]. 

## 4. Discussion

The emergence of resistance to fluoroquinolones in virtually all species of bacteria was recognized soon after the introduction of these compounds for clinical use [1, 480]. During the last several years, resistance to fluoroquinolones has remained very high among MRSA, *P. aeruginosa* and anaerobes as well as in pathogens isolated from intensive care unit-patients. More worrisome are recent reports of an overall increase in resistance to fluoroquinolones among bacteria causing community-acquired infections, such as *E. coli* and *N. gonorrhoeae*. These surveillance data demonstrate that fluoroquinolone resistance has to be associated with particular bacterial species on the one hand and patient populations on the other hand. This conclusion has been drawn by Acar and Goldstein already in 1997. These authors wrote: “The introduction of fluoroquinolones more than 10 years ago offered clinicians orally and parenterally administrable compounds with a broad spectrum of activity and therapeutic results not seen before for a wide range of infections, including complicated urinary tract infections, gastrointestinal infections, sexually tranmsmitted diseases, respiratory tract infections, and chronic osteomyelitis. Extensive use and misuse of these compounds led to the emergence and spread of resistant strains. Widely varying percentages of resistance to fluoroquinolones have been associated with particular bacterial species, clinical settings, origins of strains, geographic locations, and local antibiotic policies” [480]. Obviously, not much has changed since then; on the contrary, resistance rates increased to alarming high rates. The continued increase in fluoroquinolone resistance rates affects patient management and necessitates a change in some current guidelines for the treatment of, for example, urinary tract infections [145–147], or even precludes the use of fluoroquinolones in the treatmrnt of severe intra-abdominal infections [8] or sexually transmitted diseases [399, 435–438]. The consequences to be drawn are discussed indication-specifically above.

Although *S. pneumoniae* and *H. influenzae*, causing community acquired respiratory tract infections (CARTIs), remained highly susceptible to fluoroquinolones, 10- to 30% of *H. influenzae* and *S. pneumoniae* causing CARTIs harbored first-step-mutations in the quinolone resistance determining region conferring low-level fluoroquinolone resistance. These mutants pass susceptibility testing unnoticed and are primed to acquire high-level fluoroquinolone resistance rapidly, thus putting the patient at risk. Implementation of a fluoroquinolone therapy in patients harboring such first step mutants, in particular in elderly, immunocompromised patients, and patients with additional risk factors will likely result in the selection of resistance, paralleled by clinical failure. 

Of major concern is the association of fluoroquinolone resistance and ESBL-production in Enterobacteriaceae. One- to two thirds of Enterobacteriaceae producing extended spectrum *β*-lactamases were fluoroquinolone resistant, too, thus limiting the fluoroquinolone use in the treatment of community—as well as healthcare acquired urinary tract—and intra-abdominal infections as well as travelers' diarrhea in all those geographic areas in which fluoroquinolone resistance rates and/or ESBL-production is high. The remaining ESBL-producing or plasmid-mediated quinolone resistance mechanisms harboring *Enterobacteriaceae* were low-level quinolone-resistant, thus, being primed to acquire high-level resistance during treatment. Furthermore, fluoroquinolones like ciprofloxacin and levofloxacin select for methicillin resistance in staphylococci. Consequently, their clinical use is limited in those indications in which staphylococci are the predominant pathogens, like skin and skin structure infections. But fluoroquinolones should be used with caution even in the treatment of infections rarely caused by staphylococci like urinary tract infections, because of the MRSA selective potential, thus causing “collateral damage” [146]. 

The co-selection of fluoroquinolone resistance by *β*-lactams or aminoglycosides, and vice versa *β*-lactam- or aminoglycoside resistance by fluoroquinolones demonstrates that chemically unrelated drug classes select for drug-resistant mutants and even multidrug resistant strains, so that the emergence and spread of such strains has compromised the clinical utility of diverse antibacterials. 

Successful clones of resistant bacteria are often spread horizontally either due to poor hygiene, transfer of patients from one ward to another or from a hospital to a nursing home, as well as interregional migration and international population mobility. Thus, humans are mobile vectors of drug resistance [481]. Both, exposure of bacterial pathogens to antibacterials and environmental factors have a role in the emergence and spread of resistance. Furthermore, inappropriate antibiotic policies, poor compliance, suboptimal dosing, diagnostic and laboratory error, ineffective infection control, counterfeit or altered drugs contribute to the selection of resistance. Pleiotropic factors have an impact on the fluoroquinolone resistance epidemiology; as resistance rates vary significantly between and within countries, antibiotic prescribing must be viewed against this background of diverse processes contributing to the emergence and spread of antimicrobial drug resistance. 

## Figures and Tables

**Table 1:** Worldwide prevalence of fluoroquinolone-resistant uropathogens. Data in columns four and five represent total numbers of isolates studied; data in columns six to nine represent fluoroquinolone-resistant isolates in percent of total; figures in column ten show ESBL-positive isolates in percent of total isolates studied; figures in columns eleven and twelve represent ESBL-positive or negative fluoroquinolone-resistant isolates in percent of total numbers of ESBL-positive isolates.

Species	Country	Sampling period	*n* uUTI	*n* cUTI	HAUTI	HAUTI	CAUTI	CAUTI	ESBL pos	% FQ res.	% FQ res.	Ref.
					uUTI	cUTI	uUTI	cUTI	% of	of ESBL	of ESBL	
					% FQ res.	% FQ res.	% FQ res.	% FQ res.	total	pos.	neg	
*E. coli*	ESP	03/03 to 01/03	82	82	—	19.5	8.5	—	—	—	—	[482]
*E. coli*	GRC	01/05 to 03/06	1.936	—	—	—	2.2	—	—	—	—	[483]
*E. coli *	PRT	03/04 to 03/06	—	90	—	—	—	98	96	100	—	[120]
*E. coli*	TUR	2005 to 2006	107	—	—	—	25.2	—	—	—	—	[484]
*S. aureus*	TUR	2005 to 2006	12	—	—	—	41.7	—	—	—	—	[484]
*Enterococcus *spp.	TUR	2005 to 2006	5	—	—	—	20.0	—	—	—	—	[484]
*E. coli*	TUR	01/07 to 12/07	269	34	—	—	22.0	41.0	—	—	—	[90]
*E. coli*	TUR	—	321	290	—	—	17.0	38.0	71	7.9	92.1	[485]
*E. coli*	TUR	—	110	—	—	—	15.0	—	—	—	—	[486]
*E. coli*	LBN	2000	395	—	17.0	—	—	—	—	—	—	[487]
*E. coli*	LBN	2009	628	—	48.0	—	—	—	—	—	—	[487]
*E. coli*	IND	08/04 to 07/05	61	—	—	—	69.0	—	—	34.4	65.6	[488]
*K. pneumoniae*	IND	08/04 to 07/05	22	—	—	—	47.0	—	—	27.3	72.7	[488]
*E. coli*	IND	06/05 to 12/05	—	508	—	—	—	—	—	29.1	70.9	[489]
*K. pneumoniae*	IND	06/05 to 12/05	—	—	—	—	—	64.2	—	25.6	74.4	[489]
*Enterobacter *spp	IND	06/05 to 12/05	—	—	—	—	—	—	—	28.6	71.4	[489]
*E. coli*	IND	06/04 to 06/05	—	412	—	—	—	—	92	42.4	57.6	[94]
*K. pneumoniae*	IND	06/04 to 06/05	—	136	—	—	—	—	33	15.2	84.8	[94]
Gram-neg.* bacilli *	IND	09/01 to 12/01	—	793	—	77.5	—	—	—	71.5	28.5	[490]
Gram pos. *cocci *	IND	09/01 to 12/02	—	78	—	47.6	—	—	—	—	—	[490]
*E. coli*	IND	03/11 to 08/11	—	532	—	21	—	—	—	—	—	[491]
*E. coli*	IND	01/10 to 08/10	—	89	—	62	—	—	—	—	—	[492]
*Klebsiella *spp.	IND	01/10 to 08/10	—	32	—	48	—	—	—	—	—	[492]
*E. coli*	PAK	04/05 to 02/06	—	116	—	62.1	—	—	80.3	56.8	43.2	[493]
*E. coli*	PAK	05/07 to 09/09	—	276	—	77.2	—	—	—	—	—	[494]
*E. coli*	IRN	03/09 to 06/09	—	620	—	31	—	—	—	—	—	[495]
*K. pneumoniae*	IRN	03/09 to 06/09	—	115	—	15	—	—	—	—	—	[495]
*Enterococcus *spp.	IRN	03/09 to 06/09	—	110	—	59	—	—	—	—	—	[495]
*P. aeruginosa*	IRN	03/09 to 06/09	—	30	—	23	—	—	—	—	—	[495]
*S. aureus*	IRN	03/09 to 06/09	—	81	—	0.06	—	—	—	—	—	[495]
*E. coli*	PRK	2006	301	—	—	—	23.4	—	100	11.8	—	[89]
*E. coli*	PRK	—	688	—	—	—	—	—	71.8	12.1	87.9	[496]
*E coli*	PRK	—	—	160	—	—	—	—	71.8	23.1	76.9	[496]
*E. coli*	PRK	01/08 to 06/09	1.994	—	—	—	25.4	—	—	—	—	[497]
*E. coli*	PRK	01/01 to 12/02	232*	419*	—	—	12.7	21.2	—	—	—	[498]
*E. coli*	PRK	01/08 to 12/09	232*	419*	—	—	88	30.6	—	—	—	[498]
*E. coli *	HKG	2006 to 2008	271	—	—	—	12.9	—	—	5.2	94.8	[499]
*E. coli *	ZAF	11/05 to 10/06	87	—	—	—	11.5	—	—	2.6	97.4	[500]
*E. coli*	ZAF	11/05 to 10/06	—	366	—	17.2	—	—	—	11.9	88.1	[500]
*K. pneumoniae*	ZAF	11/05 to 10/06	17	—	—	—	11.8	—	—	31.3	68.7	[500]
*K. pneumoniae*	ZAF	11/05 to 10/06	—	182	—	31.9	—	—	—	40.6	59.4	[500]
*E. coli*	FRA	05/03 to 04/04	1.217	—	—	—	3.7	—	—	—	—	[501]
*E. coli*	ESP	2002 + 2004	5.737	—	—	—	22.7	—	—	—	—	[502]
*E. coli*	ESP	11/03 to 10/04	3.292	—	—	—	18.0	—	—	—	—	[503]
*E. coli*	RUS	1998 to 2001	456	—	—	—	4.5	—	—	—	—	[504]
*E. coli*	GBR	1999 to 2000	1.291	—	—	—	2.3	—	—	—	—	[505]
*E. coli*	ISR	1999	6.692	—	—	—	6.0	—	—	1.25	98.75	[506]
*E. coli*	USA	08/08 to 03/09	—	253	—	38	—	—	—	—	—	[506]
*E. coli*	USA	—	102	—	2.0	—	—	—	—	—	—	[507]
*E. coli*	USA	08/08 to 03/09	357	—	10.0	38.0	—	—	—	—	—	[109]
*E. coli*	NLD	1997	—	332	—	29.0	—	—	—	—	—	[111]
*Proteus *spp.	NLD	1997	—	171	—	22.0	—	—	—	—	—	[111]
*E. coli*		2004 + 2009	565	—	—	—	3.0	—	—	—	—	[508]
*E. coli*	NLD	01/04 to 12/09	—	420	—	12.0	—	—	—	—	—	[130]
*E. coli*	CHE	01/06 to 08/07	—	345	—	22.0	—	—	—	—	—	[509]
*E. coli*	CAN	09/05 to 06/06	—	283	—	19.8	—	—	—	3.5	96.5	[263]
*P. aeruginosa*	CAN	09/05 to 06/06	—	45	—	37.8	—	—	—	—	—	[263]
*K. pneumoniae*	CAN	09/05 to 06/06	—	51	—	0.0	—	—	—	1.8	98.2	[263]
*E. cloacae*	CAN	09/05 to 06/06	—	16	—	6.3	—	—	—	—	—	[263]
MSSA	CAN	09/05 to 06/06	—	20	—	20.0	—	—	—	—	—	[263]
*E. coli*	CAN	01/08 to 12/08	—	510	—	21.4	—	—	—	4.9	95.1	[264]
*K. pneumoniae*	CAN	01/08 to 12/08	—	98	—	12.2	—	—	—	3.2	96.8	[264]

uUTI: uncomplicated urinary tract infection; cUTI: complicated urinary tract infection; HA-UTI: Healthcare associated urinary tract infection; CA-UTI: community acquired urinary tract infection; ESBL: extended spectrum *β*-lactamase; Ref: reference; *total number of isolates studied in both sampling periods; ESP: Spain; GRC: Greece; PRT: Portugal; TUR: Turkey; LBN: Lebanon; IND: India; PAK: Pakistan; PRK: South Korea; HKG: Hong Kong; ZAF: South Africa; RUS: Russian Federation; USA: United States of America; NLD: The Netherlands; CHE: Switzerland; CAN: Canada.
